# PSCAN: Spatial scan tests guided by protein structures improve complex disease gene discovery and signal variant detection

**DOI:** 10.1186/s13059-020-02121-0

**Published:** 2020-08-26

**Authors:** Zheng-Zheng Tang, Gregory R. Sliwoski, Guanhua Chen, Bowen Jin, William S. Bush, Bingshan Li, John A. Capra

**Affiliations:** 1 Department of Biostatistics and Medical Informatics, University of Wisconsin-Madison, Madison, 53715 WI USA; 2 Wisconsin Institute for Discovery, Madison, 53715 WI USA; 3 Department of Biomedical Informatics, Vanderbilt University Medical Center, Nashville, 37232 TN USA; 4 Department for Population and Quantitative Health Sciences, Case Western Reserve University, Cleveland, 44106 OH USA; 5 Institute for Computational Biology, Case Western Reserve University, Cleveland, 44106 OH USA; 6 Department of Molecular Physiology & Biophysics, Vanderbilt University Medical Center, Nashville, 37232 TN USA; 7 Vanderbilt Genetics Institute, Vanderbilt University Medical Center, Nashville, 37232 TN USA; 8 Departments of Biological Sciences and Computer Science, Vanderbilt University, Nashville, 37232 TN USA; 9 Center for Structural Biology, Vanderbilt University, Nashville, 37232 TN USA

**Keywords:** Gene-level association tests, Protein 3D structures, Spatial scan approach, Risk variant detection

## Abstract

Germline disease-causing variants are generally more spatially clustered in protein 3-dimensional structures than benign variants. Motivated by this tendency, we develop a fast and powerful protein-structure-based scan (PSCAN) approach for evaluating gene-level associations with complex disease and detecting signal variants. We validate PSCAN’s performance on synthetic data and two real data sets for lipid traits and Alzheimer’s disease. Our results demonstrate that PSCAN performs competitively with existing gene-level tests while increasing power and identifying more specific signal variant sets. Furthermore, PSCAN enables generation of hypotheses about the molecular basis for the associations in the context of protein structures and functional domains.

## Background

Many whole exome or whole genome sequencing association studies, such as the National Heart, Lung, and Blood Institute Trans-Omics for Precision Medicine Program (NHLBI TOPMed) and the National Human Genome Research Institute Genome Sequencing Program (NHGRI GSP), seek to identify genes and variants that influence human complex diseases and traits [1–3]. The majority of genetic variants in the human genome are rare [4], and variants in protein-coding regions have even lower minor allele frequencies (MAFs) [5, 6]. To increase power to detect gene-level associations, set-based analyses that aggregate variants across sites within a gene are commonly employed. In particular, burden tests [7–10] for testing the mean of the genetic effects and SNP-set kernel association tests (SKAT) [11–13] for testing the variance of the effects are widely used set-based approaches. Burden tests are more powerful when the association effects are similar across the aggregated variants, while SKAT is more powerful when the effects are in opposite directions or the number of causal variants is small relative to neutral variants [14].

Despite the popularity of these gene-level association tests, their power is limited by the high background rate of neutral variants, even in causal genes. To address this issue, one approach is to only consider variants that are likely to be causal based on their functional annotations. For example, it is a common practice to include only loss-of-function variants or variants with high functional effect predictions from algorithms such as PolyPhen2 [15] and SIFT [16]. However, these scores have only modest accuracy, and scores from different tools often disagree [17]. In addition, most functional annotations are not phenotype-specific, further limiting their effectiveness in filtering neutral variants from association analysis.

An alternative approach to address the challenge posed by the high background rate of neutral variants is based on the assumption that disease-causing variants tend to cluster in specific regions of a gene or chromosome. Indeed, studies have reported clustering of disease mutations for several Mendelian and complex diseases [18–22]. For example, variants in gene LRP2 associated with autism spectrum disorder cluster mostly in a 25 kb region of the gene [22]. As a result, scan tests provide an attractive framework to search for clustered association signals in a certain genetic region [22–24]. In scan tests, a window of fixed size is moved along the length of the region, a test statistic is computed for the variants within each window, and the window with the strongest evidence of association is identified as the signal cluster.

For protein-coding variants, current scan tests ignore the functional context of the variants – 3D protein structure. Within protein structures, variants that are distant along the genome may be nearby in 3D protein space due to the process of protein folding. Clustering of tumor-derived somatic mutations has been reported in many proteins, and the identified clusters often overlap known functional regions of oncogenes and tumor suppressors [25–30]. Germline mutations also display non-random spatial patterns. For example, protein-protein interaction interfaces are enriched for disease-causing germline missense variants, but depleted for neutral missense variants [31]. A recent systematic investigation of the spatial distribution of genetic variants in human protein structures concluded that germline disease-causing missense variants are generally clustered in protein structures, whereas neutral variants exhibit a trend toward spatial dispersion [32].

In this paper, we develop PSCAN, a scan test approach that leverages the tendency of functional variants to cluster in 3D protein space. PSCAN enables us to powerfully discover disease-associated genes and identify potentially causal variants. As it is generally unknown a priori how functional variants may cluster in 3D space, our method is built upon the flexibly shaped spatial scan framework, with scan windows adaptively defined to accommodate diverse topologies of variant positions in protein space. Within each scan window, we conduct set-based tests of the mean or variance of the genetic effects. The *p*-values from set-based analysis across windows are then combined to generate a global *p*-value for evaluating the gene-level association. Furthermore, we devise and implement a search algorithm for detecting non-overlapping signal windows/regions in the gene that exhibit significant associations. The variants within the regions are referred to as signal variants, and they highlight important protein domains with potential biological functions contributing to the particular trait of interest.

Results from simulation studies and application to two real data sets suggest that PSCAN provides improved power for identifying disease-associated genes and signal variants. In particular, the PSCAN tests are substantially more powerful than existing gene-level tests in the presence of signal clusters in protein space, and they maintain similar performance in the absence of signal clusters. When applied to real data, PSCAN identifies signal variants in relevant functional protein domains, which provides valuable insights into the underlying biological mechanisms of the disease. In addition, PSCAN only requires variant-level summary statistics and is computationally efficient, which make it desirable for practical use.

## Results

### Mapping variants into 3D protein space

To obtain 3D coordinates of genetic variants in protein space, we first consider experimentally determined protein structures. If no experimental structure is available, we then consider computationally predicted structures. We extract 3D coordinates for all single chain entries in the asymmetric units of all human protein structures in the Protein Databank (PDB, http://www.rcsb.org/) [33]. For all structures, alternative atom positions are not included. Structure files with multiple models, e.g., from nuclear magnetic resonance spectroscopy, are represented by the first reported model. Because of the dynamic nature of proteins and difficulties in elucidating structures of larger proteins, some regions of proteins may be missing in available structural models or broken up into fragments across multiple models. When structural information for a particular protein is incomplete, variants that are not mapped to residues with structural information have missing coordinates. In the case of multiple structures representing different parts of a protein, it is not always possible to reliably combine the fragments into a single model on a proteome-wide scale. In the next section, we describe how we handle unmapped variants and multiple structures in defining scan windows.

Because experimentally determined structures are available for only approximately 30% of the human proteome [32], we include computationally derived models generated by the Modbase pipeline [34]. These 3D models are predicted using coordinates from experimentally determined structures of proteins closely related to the protein modeled. Considering computationally derived structures more than doubles the number of human proteins with 3D structural information. In our applications to real data, over 80% of the genes have structural models for at least part of their sequences. We demonstrate in the section “Application to the NHLBI exome sequencing project data” that computational structures are sufficiently accurate to guide our scan tests.

For experimentally determined structural models, all non-synonymous variants of interest are mapped to protein structures using the previously described PDBMap method [32]. In brief, this pipeline maps genetic variants into the mRNA transcripts they influence, then into the resulting protein sequences, and ultimately into available protein 3D structures. It first annotates the transcript-level impacts of coding variants using v82 of the Ensembl Variant Effect Predictor (GRCh37.24) [35]. These transcript sequences are then linked to the corresponding protein sequences from the Uniprot database [36]. Finally, these protein sequence variants are mapped to positions in protein 3D structures through SIFTS (Structure Integration with Function, Taxonomy and Sequence) alignments [37]. Synonymous variants could also be considered by mapping them to their positions in structure, but since they are less likely to be causal than non-synonymous variants, we do not consider them here. Computationally predicted 3D structures from Modbase are generated directly from transcript sequences, so no alignment is necessary to translate protein sequences to Modbase model coordinates.

### Defining flexibly shaped windows in 3D protein space

To perform scan tests in a 1D setting, such as along a chromosome, a window [*t*,*t*+*w*] of fixed size *w* is moved along the chromosome to define the potential cluster regions. Among all possible values of *t* and *w*, the one that produces the largest test statistic is recorded and compared to its distribution under the null hypothesis to assess the significance of association.

In 3D protein space, there are many possible approaches to defining the “window”. For example, a window can be defined as a subset of variants in a spherical [38, 39], rectangular [40], or elliptical [41] region with a predetermined angle. The major problem with such window definitions in our application is that they have a prespecified shape, while our potential disease-associated clusters may have diverse and complex shapes depending on 3D protein folding. Another challenge in traditional scan tests is that the number of windows is usually very large considering various locations and sizes of the shapes, which makes the scan test computationally intensive and subject to high penalty for multiple-testing correction.

To overcome these limitations, new spatial scan tests have been developed to allow signal regions to have flexible shapes [42–44]. In light of these methods, we propose a scan approach that can adapt to the topology of variant locations in protein space. Specifically, let *C*_1_,…,*C*_*m*_ denote the 3D coordinates of variants in the protein. For a fixed window size *w*>0, we define a graph $\mathcal {G}(w)$ with edges {(*i*,*j*):*d*_*ij*_≤*w*,1≤*i*≤*m*,*i*≤*j*≤*m*}, where *d*_*ij*_ denotes the Euclidean distance between locations *C*_*i*_ and *C*_*j*_. In this graph, the variants *i* and *j* are connected if their Euclidean distance is less than *w*. We say two variants are in the same window if a path exists between the two variants in graph $\mathcal {G}(w)$. By changing size *w*, a series of graphs and associated windows are generated. Even though *w* can take an infinite number of values, the number of possible graphs is limited because the graph $\mathcal {G}(w)$ remains the same when *w* is between two consecutive values in the sorted list of pairwise distances *d*_*ij*_’s. Moreover, although new edges will be added to the graph $\mathcal {G}(w)$ when *w* reaches the next *d*_*ij*_, the set of connected components (i.e., windows) of the graph may remain the same. Hence, the number of possible windows considered in the scan procedure is small.

**Fig. 1:**
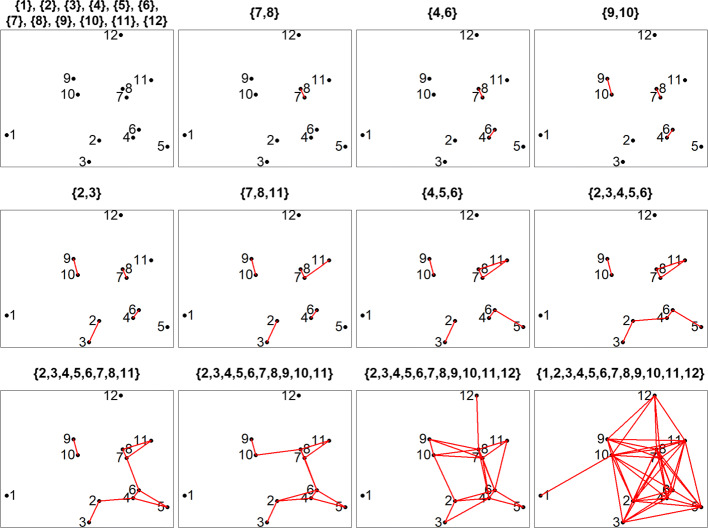
Example of window definition in 2D space. Each subfigure corresponds to a unique graph generated in the scan procedure. Windows are defined from the connected components in each graph. The newly introduced window(s) from each graph are listed in the title. In PSCAN, the coordinates are determined by the locations of variants in 3D protein space

**Fig. 2:**
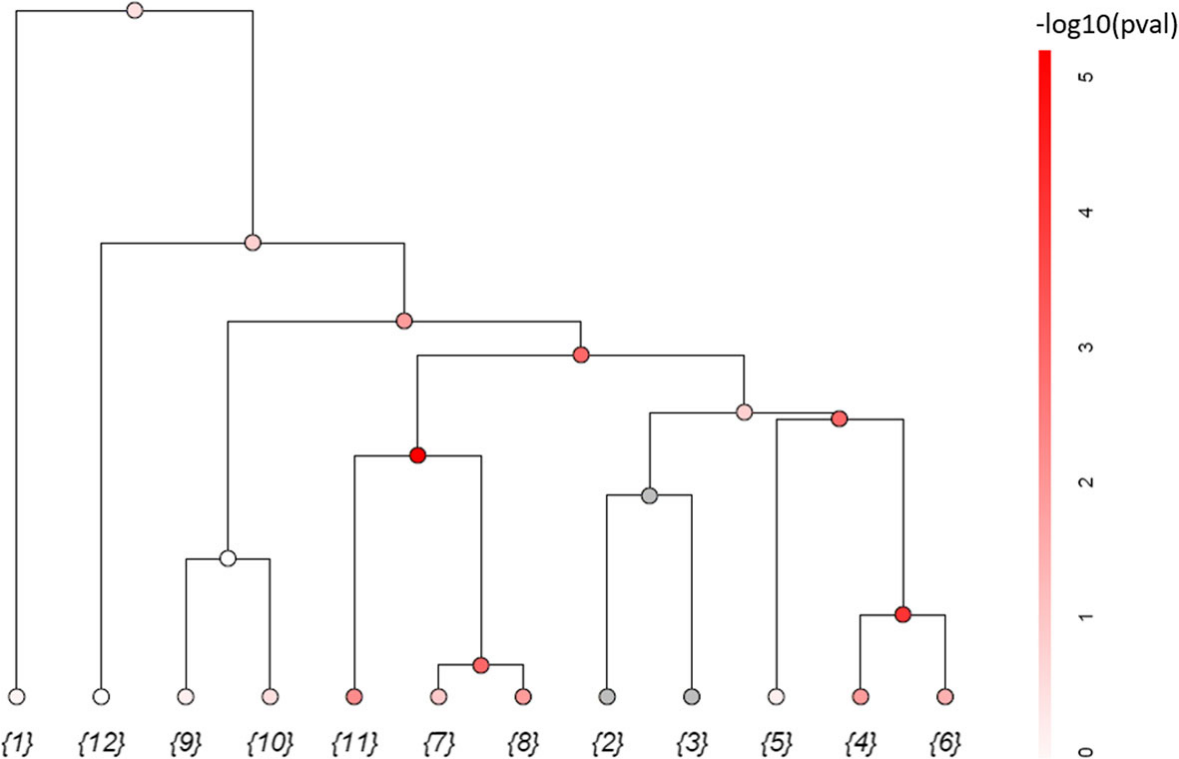
Tree representation of window formation. Each node in the tree represent a window. The terminal nodes are individual variants. The windows with gray nodes are omitted because of low cumulative minor allele counts. The other windows are colored red that reflect the magnitudes of the set-based test *p*-values

In Fig. 1, we provide a simple example in 2D space to illustrate the window definition. Suppose we have 12 variants coded by numbers 1 to 12. Although there are 66 pairwise distances, there are only 12 graphs that give different windows. From these graphs, we can derive 23 possible windows: {1}, {2}, {3}, {4}, {5}, {6}, {7}, {8}, {9}, {10}, {11}, {12}, {7,8}, {4,6}, {9,10}, {2,3}, {7,8,11}, {4,5,6}, {2,3,4,5,6}, {2,3,4,5,6,7,8,11}, {2,3,4,5,6,7,8,9,10,11}, {2,3,4,5,6,7,8,9,10,11,12}, {1,2,3,4,5,6,7,8,9,10,11,12}. The window formation can be visualized using a tree diagram in Fig. 2. Given *m* variants, this procedure generates 2×*m*−1 possible windows (represented by nodes on the tree in Fig. 2). This number is substantially smaller than that in traditional spatial scan approaches. In Additional file 1: Figure S1, we demonstrate the definition of windows in the traditional spherical-region scan approach [38, 39] using the same example. This approach produces 87 spherical windows, and the window definition would change if a different shape were adopted.

Our scan approach can detect irregular shaped clusters, whereas the traditional approaches use windows of fixed shape to capture the potential clusters. In Additional file 1: Figure S2, we show another example using variant coordinates on a fragment of the protein SORL1. In this example, our scan approach produces a window that contains several variants clustering in a banded region. This illustrates how scan windows in our approach are adaptively defined to accommodate diverse topologies of variant clusters in protein space.

As described in the previous section, a protein may have missing structural models for some regions and multiple structural models that cover different regions but cannot be reliably combined. In our scan method, we define a window set for each structure separately, create an additional window to include unmapped variants (i.e., variants in the regions missing structural models), and finally generate a global window by merging all the windows.

### Set-based tests in each window

Once the windows are defined, we test the association of variants in each window to the disease. Many set-based association tests have been proposed to aggregate variants in a certain region. In particular, burden tests create burden scores by collapsing the variants and then testing the mean effects of the burden scores. Burden tests have good power if the aggregated variants have similar effects. On the other hand, if some variants have positive effects and others have negative effects and/or if there are many neutral variants, aggregating variants can diminish association signals. In this scenario, testing the variance of the effects, as in SKAT, is more powerful than testing the mean. In light of these existing set-based association tests, we develop two tests in the PSCAN framework: PSCAN-M for testing the mean and PSCAN-V for testing the variance. These tests can be performed by using single-variant score statistics that are often available in public portals. Compared to methods that require pooling individual level data, methods based on summary statistics are more broadly applicable, better protect study participant privacy, and offer computational advantages. The details of performing burden and SKAT tests using variant-level summary statistics are provided in Methods. The analytic *p*-values of set-based tests are inaccurate if the tests involve too few minor alleles. Therefore, we do not consider windows that contain less than 10 cumulative minor alleles.

### PSCAN gene-level association tests

The goal of PSCAN is to detect if genetic variation in a gene is associated with the trait of interest (i.e., testing the gene-level null hypothesis), and if so, to identify the signal region(s) driving the association. We focus on testing the gene-level null hypothesis in this section and identifying signal regions in the next section.

The scan method searches every window and chooses the window that provides the strongest evidence of association. We let $\boldsymbol {\mathcal {W}} = \{W_{j}\}_{j=1}^{r}$ denote the set of all the possible windows defined in protein space. The natural choice of scan statistic for gene-level association test is the minimum set-based *p*-value across all windows

1 $$ Q_{\min} = \underset{W_{j} \in \boldsymbol{\mathcal{W}}}{\min} p(Q_{W_{j}}),  $$

where $p(Q_{W_{j}})$ is the *p*-value of the set-based test statistic $Q_{W_{j}}$ in window *W*_*j*_. The $Q_{W_{j}}$ is the mean test statistic in PSCAN-M or the variance test statistic in PSCAN-V. Since many windows overlap with each other, $Q_{W_{j}}$’s are strongly correlated, and the exact distribution of *Q*_min_ is hard to derive. To assess the significance of a minimum *p*-value scan statistic, we construct its empirical null distribution using Monte Carlo simulation (Methods). While simulation-based approaches can be used to incorporate the correlations and evaluate the significance of the minimum *p*-value test, they are computationally expensive, especially for the significance level required when testing all genes in the human genome.

To address this challenge in our genome-wide applications, we use the Cauchy method [45] to combine multiple set-based *p*-values across windows. Similar to the minimum *p*-value method, the Cauchy method focuses on the few smallest *p*-values. The advantage of Cauchy method over the minimum *p*-value method is that the Cauchy *p*-value combination does not require accounting for the correlation of the individual tests. In particular, the Cauchy method defines the test statistic as

2 $$ Q_{cauchy} = \sum_{W_{j} \in \boldsymbol{\mathcal{W}}} tan\left\{ \left[ 0.5-p(Q_{W_{j}}) \right] \pi \right\} /|\boldsymbol{\mathcal{W}}|,  $$

where $|\boldsymbol {\mathcal {W}}|$ is the total number of windows. The *p*-value of *Q*_*cauchy*_ can be accurately approximated by $ \frac {1}{2} - \frac {arctan \left (Q_{cauchy}\right)}{\pi }. $ The Cauchy method has recently been adopted in association analysis to combine different rare variant association tests and has been shown to have superior performance over the minimum *p*-value method in genome-wide association studies [46].

### PSCAN search algorithm for signal regions

Given a disease-associated gene, it is important to further pinpoint potentially causal variants in the gene. Partitioning protein space using windows allows us to identify local signal regions in a meaningful biological context. In the signal detection stage, we usually analyze only the handful of known disease-associated genes, so we do not need as strict a control on the nominal type I error as in the genome-wide association study. Therefore, it is feasible to adopt the minimum *p*-value scan statistic (1) and use Monte Carlo simulation to obtain the significance threshold (Methods).

We summarize the PSCAN procedure for identifying signal regions in Algorithm 1 in Methods. In particular, we first pick candidate signal regions as windows with set-based test *p*-values less than the significance threshold. Among the candidate signal regions, we use an iterative algorithm to identify multiple non-overlapping regions. In each round, we select a region that has the smallest *p*-value among all the candidate regions and remove regions that overlap with the selected region from the pool of candidates. In Fig. 2, suppose that we have candidate sets {4,6}, {4,6,5}, {7,8}, {7,8,11}, {2,3,4,5,6,7,8,11} that pass the significance threshold, among which {7,8,11} has the smallest *p*-value. The algorithm will pick {7,8,11} as a signal region in the first round and remove {7,8} and {2,3,4,5,6,7,8,11} because they overlap with {7,8,11}. Among the remaining candidates, suppose {4,6} has smaller *p*-value than {4,6,5}. The algorithm will pick {4,6} as another signal region in the second round, remove {4,6,5}, and end the search.

In the setting of scan methods in 1D, this signal detection approach can achieve asymptotic optimality [24, 47] (i.e., in reliably separating the true signal region from noise) when signals are sufficiently strong and signal regions are well separated. However, an alternative signal region identification approach has been developed to deal with situation where signals are relatively weak and/or signal regions are possibly nested [48, 49]. This procedure only removes windows that overlap by more than the pre-specified overlap fraction *f*. When *f*=1, this algorithm essentially keeps every region passing the significance threshold as the detected signal regions. In this paper, we focus on evaluating the non-overlapping-window search algorithm but our software incorporates the alternative approach (see Additional file 1: Algorithm S1).

### Simulation studies

#### PSCAN controls type I error and improves power

We carried out extensive simulations to investigate the performance of PSCAN in gene-level association testing and signal region detection and compare it with existing methods. Specifically, we compared the power of PSCAN-M and -V with 1D scan tests SCAN1D-M and -V based on the variant position on the chromosome (Methods), and standard burden and SKAT tests. The genotype and phenotype simulation strategy is detailed in Methods. In short, for each simulation, we generated genotypes for 5000 subjects following a European-ancestry demographic model, and simulated variant 3D locations and genetic effects under a wide range of signal dispersion levels and effect size distributions. We assessed the type I error rates for PSCAN gene-level association tests and signal region detection. In association testing, we set the nominal significance level *α* at 10^−4^, 10^−5^ and 2.5×10^−6^, and used 50 million replicates to estimate the empirical type I error rate under the null model; the empirical power was estimated at the significance level *α* of 10^−6^ based on 10^3^ replicates. In signal region detection, we set the nominal significance level *α* at 0.05 and 0.01, and used 10^3^ replicates.

The results for type I error rates are shown in Table 1. For testing gene-level associations, PSCAN-M and -V have properly controlled type I error. The PSCAN-V test is slightly conservative because the set-based variance test conducted in each window is conservative in the presence of rare variants [13]. The type I error rate is also protected in PSCAN-M and -V procedures for detecting signal regions.

**Table 1:** Type I error rate of PSCAN gene-level association test and signal region detection

	*α*	PSCAN-M	PSCAN-V
Gene-level association test	10^−4^	1.0×10^−4^	9.5×10^−5^
	10^−5^	9.9×10^−6^	8.8×10^−6^
	2.5×10^−6^	2.4×10^−6^	2.0×10^−6^
Signal region detection	0.05	0.049	0.048
	0.01	0.011	0.009

To evaluate power, we randomly chose 10% or 50% of variants to be causal to reflect sparse and dense signals, respectively. In addition, we considered two effect direction scenarios: (1) unidirectional effects – all causal variants increase the trait value; and (2) bidirectional effects – half of the causal variants increase the trait value, and the remaining half decrease the trait value. Finally, we considered different spatial dispersion levels of causal variants. The coordinates for the neutral variants were sampled from a standard normal distribution. The coordinates for the causal variants were sampled from a zero-mean normal distribution with standard deviation *ρ*. We simulated low, medium, or high dispersion levels by setting *ρ*=0.1, 0.25 or 1. Low *ρ* (low dispersion) places causal variants in a small region that includes almost no neutral variants, and *ρ*=1 (high dispersion) makes the causal variants completely mixed with neutral ones. Additional file 1: Figure S3 shows variant coordinates in 2D for different signal dispersion levels based on example simulated data sets.

**Fig. 3:**
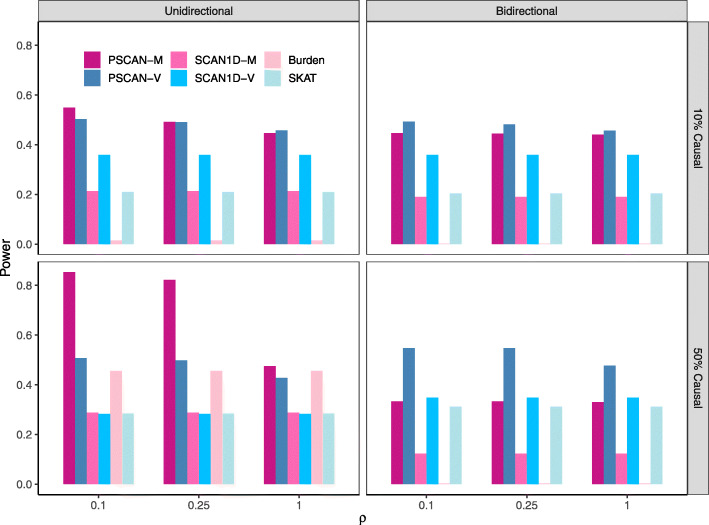
Power comparisons of PSCAN-M, PSCAN-V, SCAN1D-M, SCAN1D-V, burden and SKAT tests. Each bar represents the empirical power estimated as the proportion of *p*-values less than 10^−6^. The left panel assumes unidirectional genetic effects and the right panel assumes bidirectional effects; the upper panel assumes 10% of the variants are causal and the lower panel assumes 50% of the variants are causal. For each configuration, low (*ρ*=0.1), medium (*ρ*=0.25) and high (*ρ*=1) dispersion levels of signal variants are considered

The results under different scenarios are shown in Fig. 3. The PSCAN-M test is more powerful than PSCAN-V when the effects are unidirectional and the causal variants are spatially clustered. The PSCAN-V test becomes more powerful than PSCAN-M when the effects are bidirectional or the causal variants are spatially dispersed. In the sparse signal setting, PSCAN-M and PSCAN-V are substantially more powerful than all the other methods, even when causal variants are completely mixed with neutral ones (*ρ*=1); the SCAN1D-M and SCAN1D-V tests are more powerful than their counterparts in burden and SKAT tests. In the dense signal setting, when the effects are unidirectional, the power of PSCAN-M and PSCAN-V becomes similar to that of burden test when *ρ*=1. SCAN1D tests are less powerful than the burden test in this scenario (i.e., dense unidirectional signals evenly spread over the gene region), because they pay high penalty for testing many windows when the most effective window is the whole gene region. When the effects are bidirectional, PSCAN-V is much more powerful than the other tests regardless of *ρ*, and the power of PSCAN-M is similar to that of SCAN1D-V and SKAT tests.

#### PSCAN accurately detects simulated signal regions

**Fig. 4:**
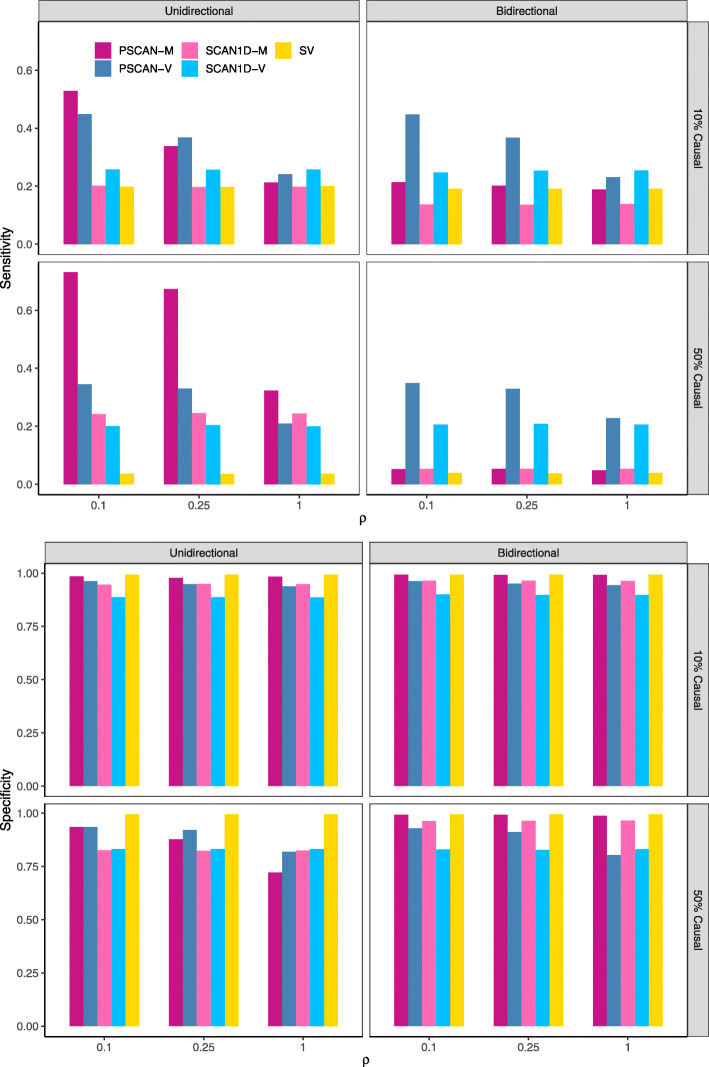
Signal detection accuracy comparisons of PSCAN-M, PSCAN-V, SCAN1D-M, SCAN1D-V, and single variant (SV) analysis. Sensitivity and specificity were calculated to measure the performance of the signal detection methods at *α*=0.05 level. In each measure, the left panel assumes unidirectional genetic effects and the right panel assumes bidirectional effects; the upper panel assumes 10% of the variants are causal and the lower panel assumes 50% of the variants are causal. For each configuration, low (*ρ*=0.1), medium (*ρ*=0.25) and high (*ρ*=1) dispersion levels of signal variants are considered

Next, we evaluated the accuracy of the PSCAN procedure for detecting potentially causal variants in simulated disease-associated genes from the scenarios described above. We compared the PSCAN procedure with the SCAN1D and single variant (SV) signal detection procedures, and quantified the performance of each method using sensitivity and specificity (Fig. 4). PSCAN-M and PSCAN-V methods outperform their counterparts, SCAN1D-M and SCAN1D-V, in sensitivity and specificity when causal variants are spatially clustered (*ρ*≠1) and have similar performance as the 1D tests when causal variants are completely mixed with neutral ones (*ρ*=1). In the setting of bidirectional effects, PSCAN-V detects more causal variants than PSCAN-M. In the setting of unidirectional effects, if the signal is dense, PSCAN-M detects more causal variants than PSCAN-V regardless of *ρ*; if the signal is sparse, PSCAN-V detects more causal variants than PSCAN-M when the causal variants tend to disperse (*ρ* increases). The SV almost always has the lowest sensitivity across all scenarios. In terms of specificity, PSCAN and SCAN1D have slightly lower values than SV, especially when the causal variants are spatially dispersed. This is not surprising, because scan methods tend to select regions that include more causal variants when their cumulative effects are large enough to overcome the inclusion of some neutral variants.

### Application to the NHLBI exome sequencing project data

#### PSCAN identifies more genes associated with lipid traits

We first applied PSCAN to sequencing and trait data from the NHLBI exome sequencing project (ESP) with the total sample size of 3,665 (Methods). We considered high-density lipoprotein levels (HDL) and triglycerides (TRIG) traits. All common and rare non-synonymous variants were included in the analysis and all methods analyzed the same set of variants. The details on data processing and acquisition of summary statistics are described in the Methods. Among 15,242 genes, 12,447 (82*%*) have protein structural models available for at least part of their sequence. The 3D coordinates for variants in protein space were derived from experimentally determined structures [33] when available (34% of the genes), otherwise from computationally predicted structures [34]. For a given gene, some variants may not have spatial coordinates due to incomplete protein structural information. Additional file 1: Figure S4 shows the distribution of the percentage of mapped variants among the 12,447 genes. As described in the section “Defining flexibly shaped windows in 3D protein space”, we created an additional window to include these unmapped variants and merged this window with other windows for mapped variants to form a global window in PSCAN.

We identified genes significantly associated with HDL/TRIG for each test at 5% false discovery rate. For most identified genes, the PSCAN gene-level tests produced more significant *p*-values compared to their burden/SCAN1D-M or SKAT/SCAN1D-V counterparts (Table 2 and Additional file 1: Figure S5). Furthermore, the PSCAN identified a more specific set of signal variants. The PSCAN *p*-value quantile-quantile (QQ) plots are well calibrated and the genomic-control lambda values are close to 1 (Additional file 1: Figure S5). PSCAN signal regions for most genes contain only a few variants; however, in NCK1, APOC3 and CYP2C9, nearly all variants are detected as signal variants. As a result, PSCAN *p*-values are not always more significant than their burden/SCAN1D-M and SKAT/SCAN1D-V counterparts for these genes.

**Table 2:** Genes significantly associated with lipid traits in at least one test on the ESP

Trait	Gene	Pval PSCAN-M	Pval PSCAN-V	Pval SCAN1D-M	Pval SCAN1D-V	Pval Burden	Pval SKAT	#SNP Total	#Signal SNP in (PSCAN-M,-V), (SCAN1D-M,-V), SV
HDL	CD36	1.2×10^−6^	2.6×10^−7^	9.0×10^−3^	3.9×10^−7^	2.7×10^−4^	1.1×10^−7^	70	(2,2), (0,48), 1
	CETP	6.9×10^−7^	2.4×10^−9^	7.0×10^−9^	1.1×10^−9^	4.9×10^−4^	9.1×10^−8^	35	(3,4), (2,3), 1
	NCK1	3.6×10^−1^	6.5×10^−6^	4.0×10^−1^	2.4×10^−6^	2.5×10^−1^	6.2×10^−6^	12	(0,12), (0,7), 1
	PCSK7	1.8×10^−6^	1.8×10^−6^	2.6×10^−6^	1.7×10^−6^	1.4×10^−1^	2.4×10^−3^	28	(1,1), (1,1), 1
TRIG	APOC3	1.5×10^−5^	1.2×10^−5^	3.6×10^−8^	1.1×10^−5^	3.9×10^−6^	3.2×10^−6^	7	(7,7), (4,4), 2
	ARSI	1.4×10^−1^	1.5×10^−5^	6.1×10^−1^	1.9×10^−1^	7.3×10^−2^	4.0×10^−2^	28	(0,9), (0,0), 0
	CYP2C9	4.4×10^−6^	4.0×10^−2^	5.1×10^−5^	4.6×10^−2^	6.4×10^−7^	1.6×10^−2^	33	(30,0), (16,0), 0
	NFATC1	3.3×10^−2^	1.4×10^−7^	5.6×10^−2^	1.5×10^−6^	1.7×10^−1^	5.9×10^−2^	35	(0,6), (0,7), 0
	PGM1	2.8×10^−4^	9.9×10^−6^	2.3×10^−1^	2.9×10^−1^	9.9×10^−1^	7.2×10^−2^	27	(3,3), (0,0), 1

The association test *p*-values and the numbers of identified signal variants at *α*=0.05/5=0.01 level are presented

#### PSCAN highlights biologically relevant signal regions in protein structures

**Fig. 5:**
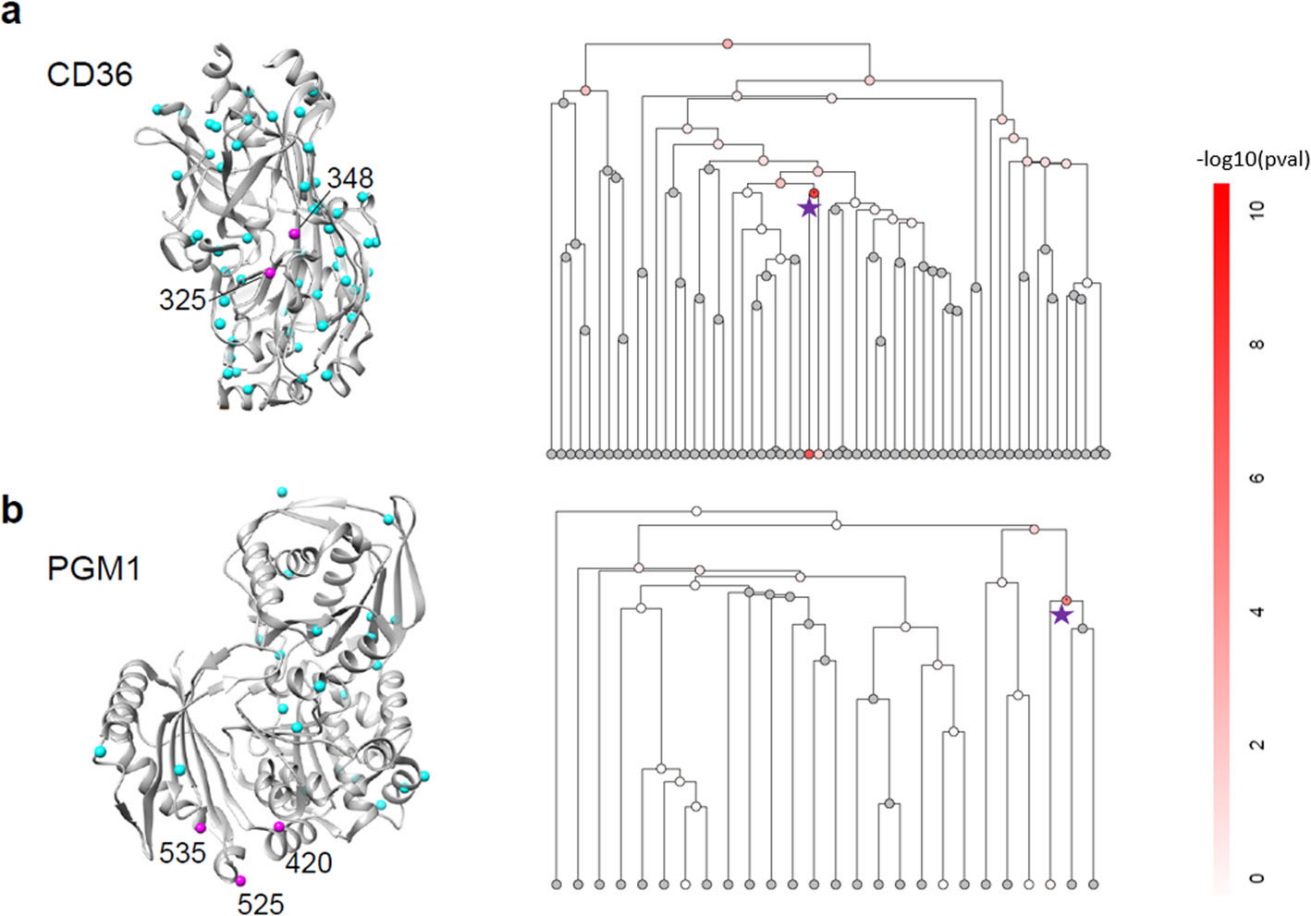
Spatial locations of significant lipid trait signal variants in CD36 and PGM1, and tree diagrams for the defined windows and associated *p*-values. **a** Two variants in CD36 (Y325*, Y348F; PDB: 5LGD) significantly associated with HDL are in a signal region that is involved in oxidized low-density lipoproteins binding. These variants are nearby in protein space, but they are 825 bp apart on the chromosome (hg19.chr7:80300449 and hg19.chr7:80301274) with two variants between them. **b** Three variants in PGM1 (Y420H, V525F, M535V; PDB: 6BJ0) significantly associated with TRIG are on the periphery of the C-terminal “domain 4” involved binding the substrate phosphate, suggesting that they may modulate activity, but not entirely disrupt binding. These variants are close in protein space, but they are more than 10 kb apart along the chromosome (hg19.1:64114301, hg19.1:64120111, hg19.1:64125260), with three other variants between them in this interval. In protein structures (left panel), the purple spheres represent signal variants and cyan spheres represent non-signal variants. The right panel shows tree diagrams representing the defined windows. The windows with gray nodes are omitted because their cumulative minor allele counts are less than 10. The other windows are colored red that reflect the magnitudes of the *p*-values from testing the mean effects. The nodes corresponding to signal regions detected by PSCAN-M are marked by purple stars

To illustrate signal windows detected by PSCAN, Fig. 5 shows the 3D protein structures, variant locations, PSCAN windows, and the associated *p*-values for two proteins: Platelet glycoprotein 4 (CD36) and Phosphoglucomutase-1 (PGM1). CD36 is a multifunctional transmembrane glycoprotein that acts as a receptor for many ligands and is involved in fatty acid metabolism, innate immunity and angiogenesis. It interacts with lipoproteins and long chain fatty acids. CD36 mutations can cause platelet glycoprotein IV deficiency [50] and increase risk for coronary heart disease [51]. PSCAN identifies two variants (Y325* and Y348F) significantly associated with HDL in CD36. These signal variants highlight a region of the protein that is involved in oxidized low-density lipoproteins binding and may also indirectly influence interactions with malarial PfEMP1 proteins [52, 53]. These two variants are adjacent to each other in protein space (Fig. 5a), but they are 825 bp apart on the chromosome (hg19.chr7:80300449 and hg19.chr7:80301274) with other two variants between them. As a result, SCAN1D-V identified a large signal window that includes many more variants than PSCAN-V for CD36, but the *p*-value associated with the window has similar level of significance (Additional file 1: Table S1).

PSCAN also identified a TRIG-associated signal region in PGM1 containing three variants (Y420H, V525F, M535V). PGM1 is an essential glucose processing enzyme that carries out the reversible conversion of glucose 1-phosphate to glucose 6-phosphate. This is a central step in many aspects of carbohydrate biosynthesis and metabolism. Missense variants in PGM1 are known to cause an inborn error of metabolism, called PGM1 deficiency, that manifests with a wide range of symptoms, including bifid uvula, cleft palate, and cardiomyopathy [54]. Mutations that cause PGM1 deficiency are found throughout the structure of PGM1 with specific hotspots in the C-terminal “domain 4” that binds the phosphate group of the substrate [55, 56]. The three variants in the signal region identified by PSCAN are all present in domain 4, but are on the periphery, suggesting that they may modulate the active site, but not entirely disrupt binding. It is also possible that they influence interactions between domain 4 and other proteins, like LDB3. These variants are nearby in protein space (Fig. 5b), but they are more than 10 kb apart on the chromosome (hg19.1:64114301, hg19.1:64120111, hg19.1:64125260), with three other variants between them in this interval. The SCAN1D tests did not identify any significant signal regions in PGM1.

#### Computationally predicted structures are sufficiently accurate for PSCAN

In the ESP data analysis, computationally predicted structures were used for 66% of the genes, since their experimentally determined structures were not available. Thus, it is essential to evaluate how the use of computationally derived models affects the performance of PSCAN tests. To this end, we computed and compared test results for proteins with both experimental and computational structural models. For direct comparison, we focused on variants that have valid coordinates in both experimentally and computationally derived structures. Although the variant coordinates and resolution are different between experimental and computational structures, our window definition procedure is likely robust to some uncertainty about variant locations since the tested windows are the same if the connected components in the series of graphs defined on pairwise distances remain unchanged. Indeed, in this analysis, on average 95% of the windows defined using experimentally determined structures were identical using the computationally predicted structures, supporting the robustness of our approach to realistic differences in resolution. Additional file 1: Figure S6 shows the comparison of the PSCAN *p*-values on experimental and computational structures. The results based on the computationally predicted structures are very similar to those based on the experimentally determined structures (Pearson correlations of 0.99). However, we note that proteins with both experimental and computational models may not be representative of all proteins with computational models. Nonetheless, the strong correlation suggests that computationally predicted structures often have sufficient resolution for use in PSCAN association analyses.

### Application to the Alzheimer’s disease sequencing project data

To further explore the potential of the PSCAN approach to identify and refine rare variant associations, we applied PSCAN-M and PSCAN-V to whole exome sequencing data from 5740 late-onset Alzheimer disease (AD) cases and 5,096 cognitively normal controls of European and Caribbean Hispanic ancestry from the Alzheimer’s Disease Sequencing Project (ADSP). Following recent work [57], we performed ancestry-stratified association analysis on missense variants with MAF < 0.05 using common covariates and combined the summary statistics from both populations using a fixed-effect meta-analysis [14] (Methods).

We identified genes significantly associated with AD for each test at 5% false discovery rate (Table 3 and Additional file 1: Figure S7). PSCAN-M identified BCAM, CBLC, CBX3, SORL1, and TREM2. Each of these genes has previously been associated with AD; however, the type and strength of evidence varies across these genes [57, 58]. In contrast, SCAN1D, burden and SKAT tests that did not consider structural information only identified BCAM, CBLC and TREM2 as associated with AD.

**Table 3:** Genes significantly associated with AD in at least one test on the ADSP

Gene	Pval PSCAN-M	Pval PSCAN-V	Pval SCAN1D-M	Pval SCAN1D-V	Pval Burden	Pval SKAT	#SNP Total	#Signal SNP in (PSCAN-M,-V), (SCAN1D-M,-V), SV
BCAM	1.2×10^−9^	8.1×10^−10^	1.6×10^−9^	5.3×10^−10^	1.4×10^−7^	8.4×10^−10^	81	(2,2), (5,5), 2
CBLC	7.3×10^−7^	2.0×10^−7^	4.1×10^−8^	1.7×10^−7^	8.1×10^−7^	4.3×10^−8^	36	(8,8), (5,5), 1
CBX3	1.2×10^−5^	4.1×10^−5^	9.7×10^−5^	4.3×10^−5^	1.9×10^−5^	4.1×10^−5^	6	(4,4), (5,2), 1
SORL1	1.2×10^−5^	5.5×10^−4^	3.2×10^−5^	4.8×10^−4^	2.1×10^−5^	2.2×10^−2^	214	(34,1), (60,7), 0
TREM2	2.5×10^−11^	8.4×10^−11^	3.0×10^−11^	3.2×10^−8^	7.8×10^−11^	3.6×10^−10^	44	(2,1), (27,23), 1

The association test *p*-values and the numbers of identified signal variants at *α*=0.05/5=0.01 level are presented

**Fig. 6:**
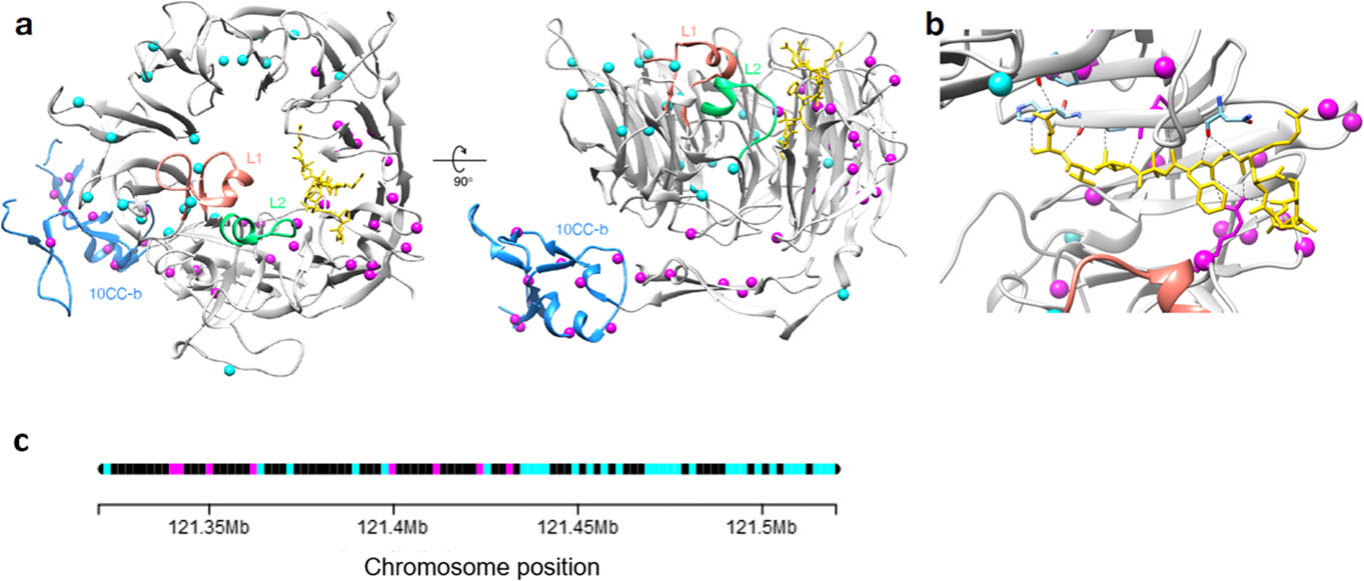
Spatial and chromosome locations of significant Alzheimer’s disease signal variants in SORL1. PSCAN-M identified an AD signal region of 34 variants (out of a total of 214 considered) in SORL1. **a** SORL1’s structure contains a ten-bladed beta-propeller domain with a large tunnel at the center that binds peptides (PDB: 3WSY), top view (left) and side view (right). SORL1 binding of peptides, including amyloid-beta, is mediated by the L1 (dark orange) and L2 (green) loops from different blades near to the entrance of propeller binding tunnel. The dynamic 10CC domain wraps around the propeller upon binding; the 10CC-b region is colored blue. Signal region variants (purple spheres) cluster in 3D space in two functional sub-regions of SORL1: one side of the peptide binding tunnel and the 10CC region. Non-signal variants are indicated by cyan spheres. **b** Many of the signal variants are in close proximity to the ligand (yellow sticks); the side-chains of residues in contact with the ligand are shown as sticks. **c** Positions of the SORL1 variants on the segment of chromosome 11 for the SORL1 gene. Signal and non-signal variants are in purple and cyan, respectively

Furthermore, the structure-aware PSCAN tests identified sets of signal variants that highlight coherent functional sub-regions of the proteins. For example, PSCAN-M identified an AD signal region of 34 variants (out of a total of 214 considered) in SORL1, sortilin-related receptor (Fig. 6 and Table 3). SORL1 is a sorting receptor protein involved in the intracellular trafficking of many peptides with propensity for beta-sheet formation, including amyloid-beta precursor protein and amyloid-beta itself [59]. SORL1 contains a ten-bladed beta-propeller domain, called Vps10p, with a large tunnel at the center that binds peptides and a dynamic 10CC domain that wraps around the propeller (Fig. 6a) [59]. SORL1 has been associated with AD through both genetic and biochemical studies [57]. However, the mechanisms underlying this association are not fully understood, and there is great interest in prioritizing genetic variants of unknown significance in SORL1 [60].

The variants in the signal region cluster in 3D space in two functional sub-regions of the SORL1 protein: the peptide binding tunnel and the dynamic 10CC region (Fig. 6a). SORL1 peptide binding is mediated by two loops (L1 and L2) from different blades near to the entrance of propeller binding tunnel. The signal region includes variants in and near the L2 loop on the side of the binding tunnel nearest to the likely location of amyloid-beta binding (Fig. 6b). The signal region also contains many variants in the flexible 10CC domain, in particular in 10CC-b, which exhibits large conformational change when peptide binding occurs. This suggests that genetic variation in these sub-domains may modulate binding activity in ways that are functionally relevant to the development of AD.

The chromosome positions of the 34 signal variants are listed in Additional file 1: Table S2 and displayed in Fig. 6c. These variants are scattered across a wide region of over 80 kb on the chromosome (position hg19.chr11:121340744 to hg19.chr11:121421364), with 24 other non-signal variants in the interval. The relative positions of these variants on a fragment of the protein and the associated PSCAN window are shown in Additional file 1: Figure S2. These 34 variants form a banded cluster in protein space. This example illustrates the flexibility of our windowing approach to find 3D signal regions beyond simple predefined shapes and that reflect biological domains that are not obvious from the linear sequence context.

## Discussion

In this paper, we propose protein-structure-based scan (PSCAN) methods to detect the existence and the locations of trait-associated signal regions in protein space. Through extensive simulations, we show that the proposed scan tests properly control the type I error rate and achieve substantially higher power compared to standard burden and SKAT tests, as well as 1D scan tests based on chromosome location. We also show that our scan procedures accurately select true signal regions and estimate their locations in 3D protein space. Furthermore, our simulation studies demonstrate that the PSCAN-M method performs better than the PSCAN-V method when the variants in the signal region have similar effects, while the trend is reversed when the effects are in different directions, or when causal variants are mixed with a large number of neutral variants in signal regions.

We applied PSCAN to whole exome sequencing data from the NHLBI ESP and the ADSP. Using protein structural information from experimentally and computationally derived 3D models, we identified several genes associated with HDL and TRIG levels and with late onset AD. Comparing the association analysis for PSCAN to burden and SKAT tests confirms the power of PSCAN to identify additional associations. Analyzing signal regions in their structural context revealed that the variants identified in these genes often cluster in functionally relevant regions of protein 3D space, while being distant along the linear protein sequence. Furthermore, considering the variants’ structural context enabled the generation of hypotheses about the molecular mechanisms underlying their associations with traits.

Our structurally guided approach for signal region detection is one of PSCAN’s main innovations. Protein structures provide the functional context for protein-coding genetic variation. Nonetheless, popular genetic association analysis methods that aggregate variants focus on the genomic context of variants of interest and do not account for biologically relevant 3D structural relationships. Thus, as we demonstrate here, the incorporation of protein-structure derived information is a powerful addition to commonly used set-based association tests. However, defining spatial windows for scan tests poses several challenges, most notably how to allow for flexibly shaped windows without requiring testing of a prohibitively large number of windows. We address this challenge by adapting a graph-based approach that creates graphs based on distances between variants and uses the connected components of the graph to define the windows of variants for testing. Ultimately, the total number of windows considered is only about twice the number of variants.

We also demonstrate that protein structural information from computationally derived models is often sufficiently accurate for PSCAN. This is a critical finding, since it substantially increases the number of proteins to which PSCAN can be applied. While only approximately 25% of human proteins have experimentally derived structures, more than 75% of human proteins have computational models of at least part of their structure available [32, 34]. As demonstrated by our application to the ESP data, sufficient structural information is available to enable the broad application of PSCAN.

PSCAN can be further extended in several aspects. (1) The development of a unified test that combines the PSCAN-M and PSCAN-V to achieve more robust power across different patterns of genetic effects would simplify their application. (2) Modeling of the uncertainty and flexibility of variant 3D locations in the scan procedure and imputation of the location of the unmapped variants would further expand and improve the scope of the PSCAN approach. (3) Leveraging additional structural information beyond the orientation and Euclidean distance between variants in 3D could also help identify more biologically relevant signal clusters. For example, the use of alternative structurally defined distance measures that incorporate information about residue-level interaction networks could capture the likelihood of functional interactions between different protein positions. (4) Finally, the application of PSCAN to structural models of protein-protein interaction interfaces and protein complexes could identify associations by integrating variants across multiple different proteins.

With the increasing number of large-scale whole exome/genome sequencing studies, incorporating external functional information into association analyses is critical to further boost discovery power and facilitate biological interpretation of results. In this paper, we focus on protein-coding variants and use 3D protein structure to detect clusters of risk variants. Other variant annotations could be readily integrated with structural information in our PSCAN framework. For example, variants could be weighted based on their evolutionary conservation or predicted effect on protein structure and stability. Integrating functional annotations as variant weights has improved power in traditional SNP-set tests. For genes with no protein structural data, we suggest using other potentially informative variant annotations. As more information about the 3D structure of genome becomes available [61, 62], our method is potentially useful for association analyses of non-coding variants by leveraging 3D chromatin structures inferred from HiChIP, ChIA-PET, and related technologies.

## Conclusions

The PSCAN method developed here is a powerful new approach for integrating the growing amounts of protein structure data into tests for finding and interpreting genetic associations. Continued development of scan tests for analysis of sequencing data will enable further novel discoveries of variants and protein regions associated with human traits and diseases. To facilitate the broad application of this approach, we provide the PSCAN R package for applying the method to whole exome and genome sequencing data.

## Methods

### Set-based association tests

Suppose ***β***=(*β*_1_,…,*β*_*m*_) are the genetic effects for the *m* variants in a given gene. We can obtain variant-level score statistics ***U***=(*U*_1_,…,*U*_*m*_) for testing *β*_1_=…=*β*_*m*_=0 and their covariance estimates ***V*** via standard regression methods [10, 63–66]. For each window *W*_*j*_, we let ${\boldsymbol {\beta }}_{W_{j}}$ denote the subvector of ***β*** for the variants contained in that window, ${\boldsymbol {U}}_{W_{j}}$ denote the corresponding subvector of ***U***, and ${\boldsymbol {V}}_{W_{j}}$ denote the corresponding submatrix of ***V***. We construct the statistic for testing $\phantom {\dot {i}\!}H_{0}: {\boldsymbol {\beta }}_{W_{j}} =\boldsymbol {0}$ in window *W*_*j*_ as

$$Q_{W_{j}} = (\boldsymbol{S}^{\mathrm{T}}_{W_{j}} {\boldsymbol{U}}_{W_{j}})^{2} /(\boldsymbol{S}^{\mathrm{T}}_{W_{j}} {\boldsymbol{V}}_{W_{j}} \boldsymbol{S}_{W_{j}}), $$

where $\boldsymbol {S}_{W_{j}}$ is a vector that includes the weights for variants in window *W*_*j*_. If we want to combine the variant-level statistics by simple summation of each component, then we set $\boldsymbol {S}_{W_{j}} = (1, \ldots, 1)$. If we want to up-weight rare variants, we can calculate $\boldsymbol {S}_{W_{j}}$ according to the MAFs of variants as described in the Madsen–Browning burden test [8]. Under the null hypothesis that no variants in the window *W*_*j*_ are associated with the trait, $Q_{W_{j}}$ follows $\chi _{1}^{2}$ distribution and its *p*-value can be computed analytically.

Trait-associated variants in a window may have effects in different directions and many neutral variants may be also present in the window. The mean scan test has low power in this scenario because the association signal is diluted by aggregating bi-directional effects and mixing in background noise. For this scenario, we employ the quadratic statistic for testing the variance. In this variance test, ${\boldsymbol {\beta }}_{W_{j}}$ is assumed to be a vector of random variables sampled from a zero-mean normal distribution with variance $\tau _{W_{j}}$. Testing $\tau _{W_{j}} = 0$ is equivalent to testing ${\boldsymbol {\beta }}_{W_{j}} = \boldsymbol {0}$. The variance test statistic takes the form

$$Q_{W_{j}} = {\boldsymbol{U}}^{\mathrm{T}}_{W_{j}} {\boldsymbol{\Omega}}_{W_{j}} {\boldsymbol{U}}_{W_{j}}. $$

The ${\boldsymbol {\Omega }}_{W_{j}}$ is a diagonal matrix with each diagonal element being the weight for each variant within window *W*_*j*_. Following SKAT [13], we set the weight based on the MAF through a Beta density function Beta(MAF; 1, 25); other weighting schemes can be readily adopted as well. Under the null hypothesis, $Q_{W_{j}}$ follows the mixture $\chi ^{2}_{1}$ distribution $\sum _{k=1}^{m_{j}} \lambda _{k} \chi ^{2}_{1,k}$, where *m*_*j*_ is the number of variants in window *W*_*j*_, *λ*_*k*_ is the *k*^th^ eigenvalue of ${\boldsymbol {V}}_{W_{j}}^{1/2} {\boldsymbol {\Omega }}_{W_{j}} {\boldsymbol {V}}^{1/2}_{W_{j}}$, and $\chi ^{2}_{1,1}, \ldots, \chi ^{2}_{1,m_{j}}$ are independent $\chi ^{2}_{1}$ random variables. The *p*-value can be accurately computed using Davies method [67].

### Minimum *p*-value scan statistic

We use the Cauchy method to combine *p*-values across windows and assess gene-level association (Results) because it is computationally fast. Previous research has shown that the Cauchy method can properly control the type I error regardless of correlations of individual tests when *p*-value is small (<10^−4^, as in genome-wide association studies) [45, 46]. However, the Cauchy method can have a slight inflation in the type I error for large *p*-values. Therefore, in the candidate gene analysis where only a handful of genes are tested, using Monte Carlo simulation to evaluate the significance of minimum *p*-value scan statistic is a more robust approach.

To assess the significance of minimum *p*-value scan statistic, we construct its empirical null distribution using Monte Carlo simulation. To be specific, we repeatedly generate ***U***^∗^ from the *m*-variate normal distribution with mean ***0*** and covariance ***V*** and recalculate the minimum *p*-value scan statistic $Q^{*}_{\min }$. These simulated $Q^{*}_{\min }$’s are used to construct the empirical null distribution for the observed scan statistic *Q*_min_. The *p*-value of the minimum p statistic is the proportion of the simulated $Q^{*}_{\min }$’s less than the observed statistic *Q*_min_.

### PSCAN signal region search algorithm

Suppose we want to control the type I error at *α* level in the signal region detection. The PSCAN search algorithm is summarized as follows. 
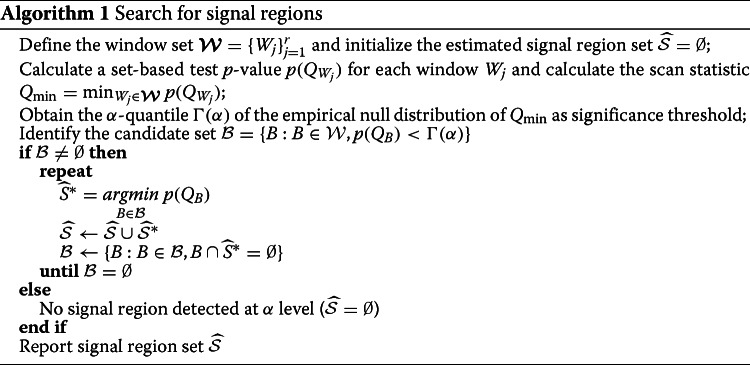


### SCAN1D method

We implemented the 1-dimensional scan (SCAN1D) method based on the variant position on the chromosome and compared its performance with PSCAN in gene-level association testing and signal variant detection. For a given gene with length *L*, we consider several window sizes: *L*/2, *L*/4, *L*/8, *L*/12, *L*/16, and *L*/20. Windows are formed by moving the interval of a given size at the skip length of *L*/40 along the gene region on the chromosome. Similar to PSCAN, a gene-based test is performed in each window and gene-level association is evaluated by combining all *p*-values across windows using the Cauchy *p*-value combination method. We refer to the SCAN1D tests of mean and variance as SCAN1D-M and SCAN1D-V, respectively. The signal region search is conducted in the same way as PSCAN. Hence, SCAN1D and PSCAN only differ in the window definition.

### Simulation strategy

For all simulations, we generated 10,000 haplotypes of length 100 Mb under a calibrated coalescent model to mimic a sample of the European population [68]. We used these haplotypes to form the genotypes of 5,000 subjects. To simulate the genotypes for a dataset, we randomly selected a 3 kb region. All variants (including common and rare) in the region were included in the analysis. The phenotypes under the null were sampled based on a linear regression model *Y*_*i*_=0.3*Z*_*i*_+*ε*_*i*_, where *Z*_*i*_ is a covariate simulated from a standard normal distribution, and *ε*_*i*_ is the standard normal error. The phenotypes under the alternative were sampled based on the linear regression model *Y*_*i*_=*β*_1_*G*_*i*1_+…+*β*_*s*_*G*_*is*_+0.3*Z*_*i*_+*ε*_*i*_, where *G*_*ij*_’s are genotypes of randomly selected casual variants and *β*_*j*_’s are the genetic effects for these casual variants. We set the effect size of the causal variant *j* to *c*|*log*_10_*MAF*_*j*_| so that low-frequency variants are not dominated by the effects of common variants. To assess the power of the gene-level tests, we set *c*=0.3 when 10% of the variants are causal and *c*=0.15 when 50% of the variants are causal so that the power of the most powerful test is reasonably high at the 10^−6^ level. To assess the accuracy of the signal region detection, we set *c*=0.25 when 10% of the variants are causal and *c*=0.1 when 50% of the variants are causal so that the sensitivity of the most powerful signal detection approach is reasonably high at the 0.05 level. We used MAF-based variant weights of $1/\sqrt {\text {MAF(1-MAF) }}$ in the PSCAN-M and burden tests and weights of Beta(MAF; 1, 25) in PSCAN-V and SKAT.

### NHLBI ESP data and analyses

To demonstrate the utility of PSCAN, we applied the method to analyze data from the NHLBI ESP [69]. The ESP consists of multiple whole exome sequencing studies, each of which is focused on a different trait: three studies of subjects with extreme values of relevant quantitative traits—body mass index, low-density lipoprotein levels, and blood pressure, one case-control study of myocardial infarction, and one case-only study of stroke. In addition to these five studies, the deeply phenotyped reference study took measurements of a set of core phenotypes on randomly sampled subjects. We used sequence and trait data from these six studies. DNA samples were sequenced on the Roche NimbleGen SeqCap EZ or Agilent SureSelect Human All Exon 50 MB at the University of Washington and the Broad Institute. We adopted the variant calling and quality control procedures as described in a previous publication [70]. We considered a total of 15,242 genes with cumulative minor allele counts ≥ 10.

After excluding subjects with sex mismatch or relatedness [63], there were 1702 African American subjects and 1963 European American subjects in our analysis. For each study, we performed association analysis for the two race groups separately using SCORE-SeqTDS [63] and obtained the variant-level score statistics and covariance estimates. We then combined these summary statistics across race groups and studies in a fixed-effects meta-analysis [14]. In the association model, we adjusted for several covariates including principal components for ancestry, age, age ^2^, gender, study cohort, and sequencing targets. As in the simulation studies, we performed PSCAN-M and burden tests with variant weights of $1/\sqrt {\text {MAF}(1-\text {MAF})}$, and PSCAN-V and SKAT tests with variant weights of Beta(MAF; 1,25).

### ADSP data and analyses

The ADSP study design, variant calling, and variant annotation are described in detail elsewhere [71–73]. We accessed whole-exome sequencing data on 5,740 late-onset AD cases and 5,096 cognitively normal controls from the ADSP Discovery phase. For comparison to existing approaches, we followed the modeling strategy outlined in Model 0 of Bis et al. [57], which included adjustments for sequencing center and ancestry-based principal components. We examined missense variants exclusively and did not include insertion-deletion polymorphisms. Variants were filtered using a MAF < 0.05, and genes were required to contain more than one variant and have a cumulative minor allele count ≥ 10. A total of 14,818 genes met this criteria, 12,730 (86%) of which has some degree of protein structural information, with 3,967 (27%) having experimentally-derived protein structures.

### Software implementations and computation time

We have implemented our method in an R package PSCAN. The PSCAN gene-level association tests do not require numerical simulations to evaluate the significance and extremely fast. In the analysis of ESP data, PSCAN takes readily available summary statistics as input and the computation time for testing 15,242 genes is less than 2 h on an IBM HS22 machine. The signal region detection requires Monte Carlo simulation to generate the null distribution of minimum *p*-value scan statistic and the computation time depend on the number of simulations and the number of variants. In our real data analysis, we used 5000 simulations in detecting signal regions at *α*=0.01 level. The PSCAN-M procedure for signal region detection takes few seconds and runs faster than PSCAN-V because numerical approximation is needed to obtain the variance test *p*-value. The PSCAN-V computation time ranges from 5 s (for APOC3 gene with 7 variants) to 4 min (for CD36 gene with 70 variants).

## Supplementary information

**Additional file 1** Supplementary material with PSCAN alternative signal region search algorithm S1, figures S1-S7, and tables S1-S2.

**Additional file 2** Review history.

## Data Availability

The ESP datasets analyzed during the current study are available in the dbGaP repository (ARIC: phs000280.v7.p1; CARDIA: phs000285.v3.p2; CHS: phs000287.v7.p1; FHS: phs000007.v31.p1; JHS: phs000286.v6.p2; MESA: phs000209.v13.p3; WHI: phs000200.v12.p3). ADSP variant summary data can be found at the NIA Genetics of Alzheimer ’s Disease Data Storage site (https://www.niagads.org) under accession number NG00065. The code to reproduce simulation studies presented in the paper is available at https://github.com/tangzheng1/PSCAN2020[74]. PSCAN R package is available under MIT license at https://github.com/tangzheng1/PSCAN[75]. The version used in this study (PSCAN v1.0.0) is available at 10.5281/zenodo.3743918[76].

## Abbreviations

3D: 3-Dimensional
ADSP: Alzheimer’s Disease Sequencing Project
HDL: High-Density Lipoprotein
MAF: Minor Allele Frequency
NHGRI: National Human Genome Research Institute
NHLBI ESP: National Heart, Lung, and Blood Institute Exome Sequencing Project
PDB: Protein Databank
PSCAN: Protein-structure-based SCAN method
PSCAN-M: PSCAN for testing the Mean
PSCAN-V: PSCAN for testing the Variance
QQ: quantile-quantile
SCAN1D: 1-Dimensional SCAN method on chromosome
SKAT: SNP-set Kernel Association Test
TRIG: Triglycerides
