# Supplementary material for: PSCAN: Spatial scan tests guided by protein structures improve complex disease gene discovery and signal variant detection
Source: Genome Biol. 2020 Aug 26;21:217. doi: 10.1186/s13059-020-02121-0 (PMC7448521; doi:10.1186/s13059-020-02121-0)
Supplement: Supplementary file 1 — Additional file 1 Supplementary material with PSCAN alternative signal region search algorithm S1, figures S1-S7, and tables S1-S2. [file 13059_2020_2121_MOESM1_ESM.pdf]

PSCAN: Supplementary Material

PSCAN alternative signal region search algorithm

The PSCAN search algorithm presented in Algorithm 1 in the main text returns non-overlapping windows with significant signals. This approach is asymptotically optimal in 1D when signals are sufficiently strong and signal regions are well separated. Furthermore, in association studies, it is usually desirable to identify as specific a set of variants as possible. However, there may be situations in which signals are weak and/or signal regions are possibly nested. To support this case, we implemented an algorithm that allows the user to set the tolerated overlap between reported sets as described below.

Suppose we want to control the type I error at α level in the signal region detection. The alternative search algorithm is summarized as follows.

Algorithm S1 Search for signal regions

Define the window set $\boldsymbol{\mathcal{W}} = \{W_j\}_{j=1}^{r}$ and initialize the estimated signal region set $\widehat{\mathcal{S}} = \emptyset$;
Calculate a set-based test p-value $p(Q_{W_j})$ for each window W_j and calculate the scan statistic $Q_{\min} = \min_{W_j \in \boldsymbol{\mathcal{W}}} p(Q_{W_j})$;
Obtain the α-quantile $\Gamma(\alpha)$ of the empirical null distribution of $Q_{\min}$ as significance threshold;
Identify the candidate set $\mathcal{B} = \{B : B \in \mathcal{W}, p(Q_B) < \Gamma(\alpha)\}$
if $\mathcal{B} \neq \emptyset$ **then**
  repeat
    $\widehat{S}^* = \underset{B \in \mathcal{B}}{argmin}\ p(Q_B)$
    $\widehat{\mathcal{S}} \leftarrow \widehat{\mathcal{S}} \cup \widehat{S}^*$
    Remove all the regions that overlap by more than the prespecified overlap fraction f with $\widehat{S}^*$ from the candidate set, that is, update the candidate set as $\mathcal{B} \leftarrow \{B : B \in \mathcal{B}, |B \cap \widehat{S}^*|/|B| \leq f\}$
  until $\mathcal{B} = \emptyset$
else
  No signal region detected at α level ($\widehat{\mathcal{S}} = \emptyset$)
end if
Report signal region set $\widehat{\mathcal{S}}$

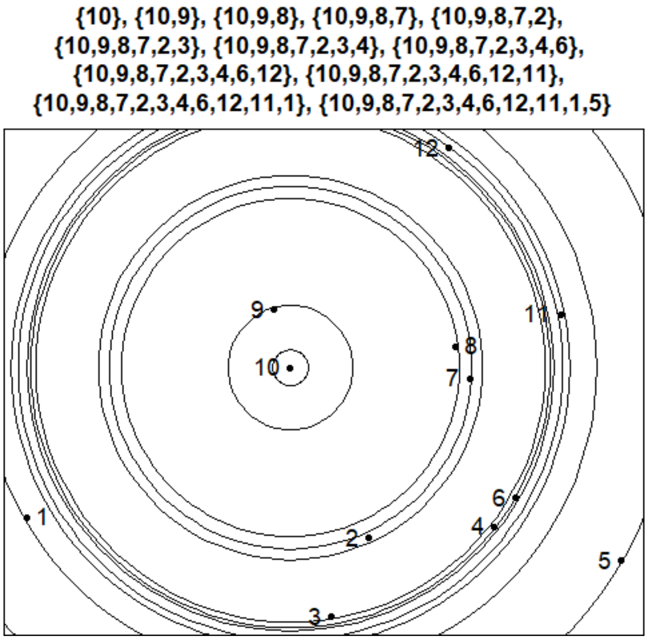

Fig. S1 Window definition in the traditional spatial scan approach using spherical regions. The node coordinates are the same as those in Fig. 1. This figure shows the 12 windows centered at node 10 and nodes included in each window are listed in the title. Windows centered round other nodes can be defined in the same fashion. In total, there are 87 unique windows defined using this traditional scan approach.

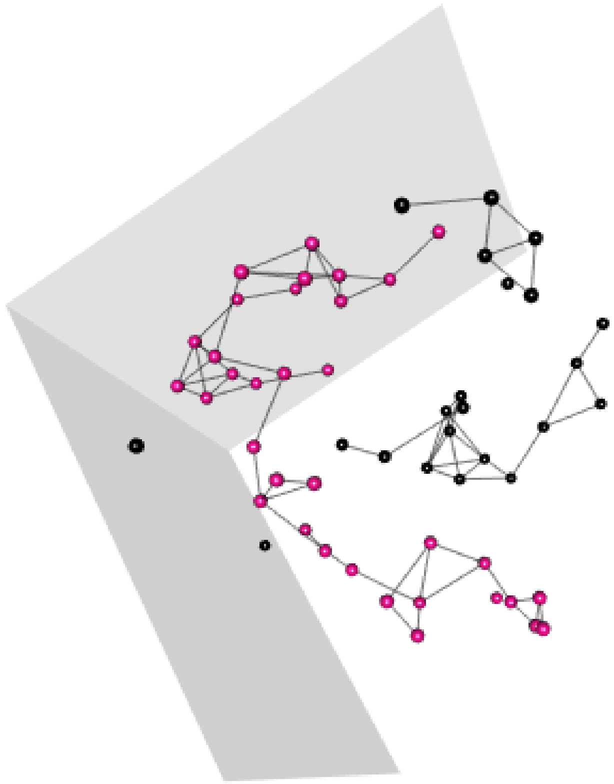

Fig. S2 ADSP variants mapped to a fragment of the protein SORL1. This graph illustrates windows defined based on the variants i and j being connected if their Euclidean distance is less than 13 Å. In our analysis of Alzheimer's disease, the signal window (purple) derived from this graph has a banded shape and includes 34 variants. This illustrates the flexibility of our window definition approach. Fig. 6 shows these variants in the context of the protein structure.

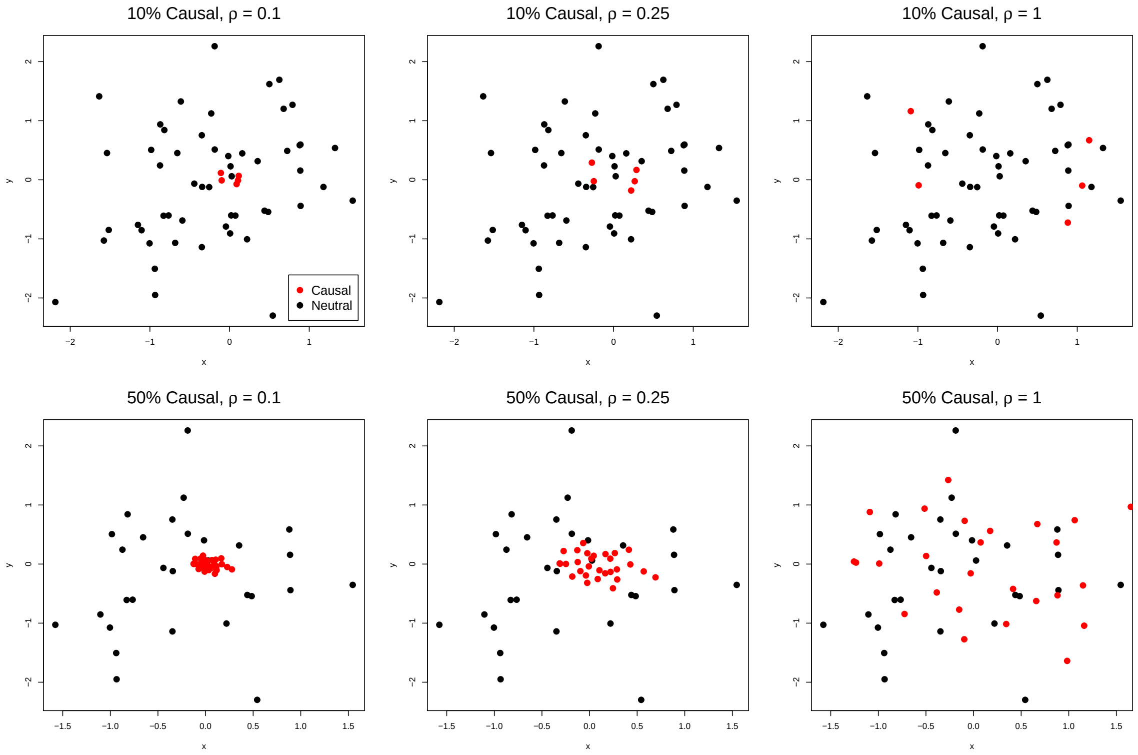

Fig. S3 Positions of variants for different signal dispersion levels under sparse (10% causal) and dense (50% causal) signal settings. The coordinates in each configuration are from an example simulated dataset.

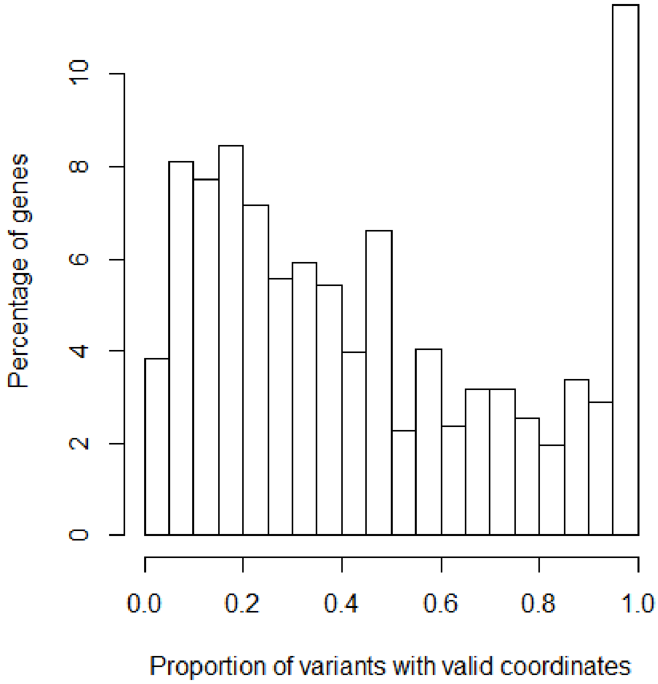

Fig. S4 Proportion of variants with valid coordinates in the corresponding protein space We considered 12,447 genes in ESP that have structural information.

(a) Trait: HDL

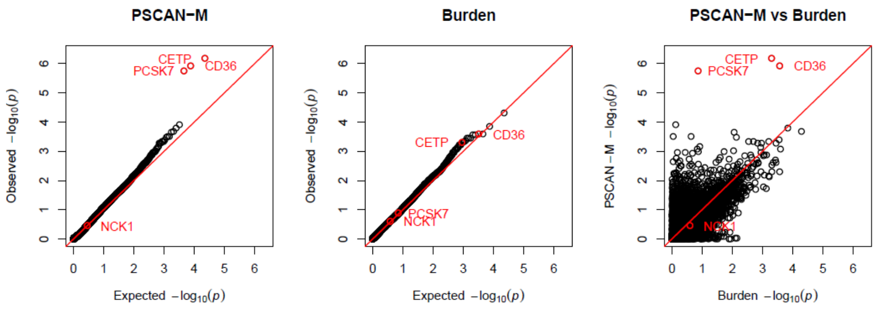

(b) Trait: TRIG

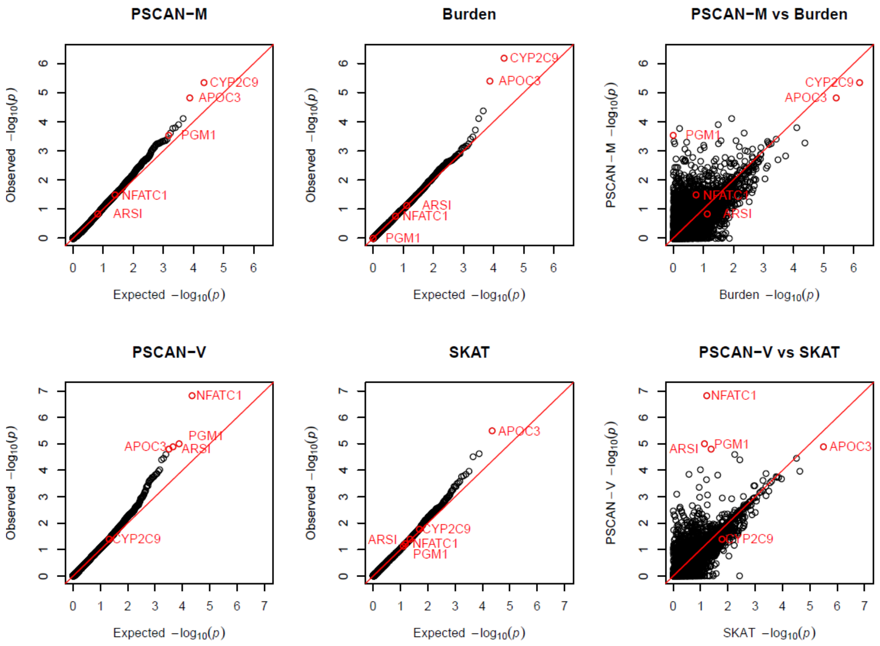

Fig. S5 Meta-analysis of the studies in NHLBI ESP for HDL and Triglycerides (TRIG). The left panel shows p-value QQ plots for the proposed tests (PSCAN-M or PSCAN-V); the middle panel shows the p-value QQ plots for the existing gene-level tests (Burden or SKAT); the right panel contains scatter plots comparing the PSCAN tests and the counterparts in existing methods. The genes significantly associated with the lipid trait in at least one test are highlighted in red.

(a) Trait: HDL

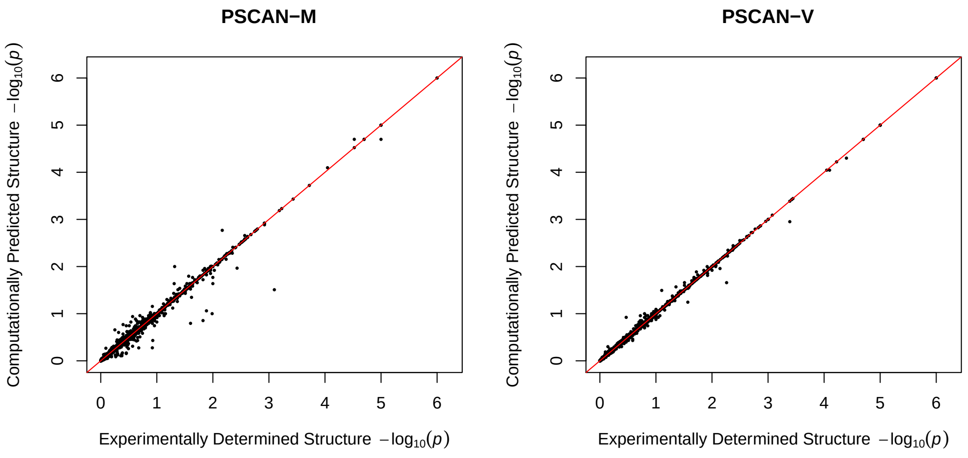

(b) Trait: TRIG

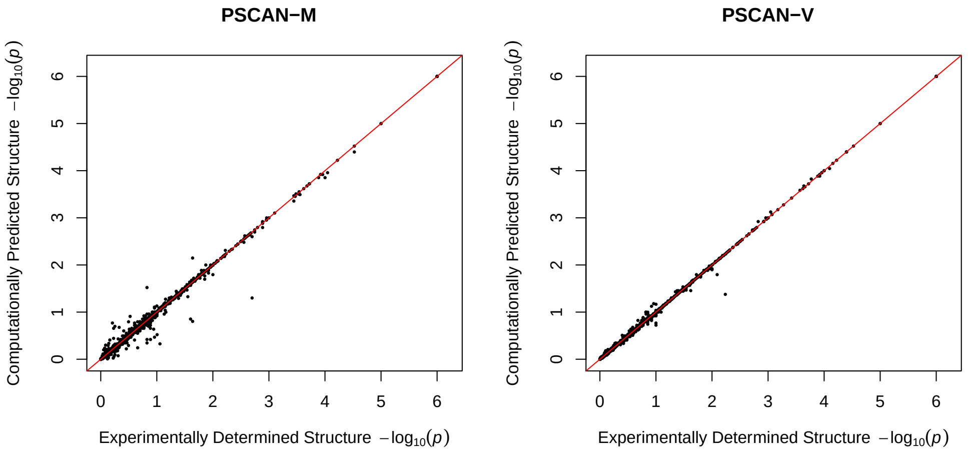

Fig. S6 Comparison between experimentally determined and computationally predicted structure in PSCAN tests. We focused on variants that have coordinates in both structures and performed PSCAN-M and PSCAN-V tests using either version of the coordiantes. The results are extremely correlated (Pearson correlations of 0.99).

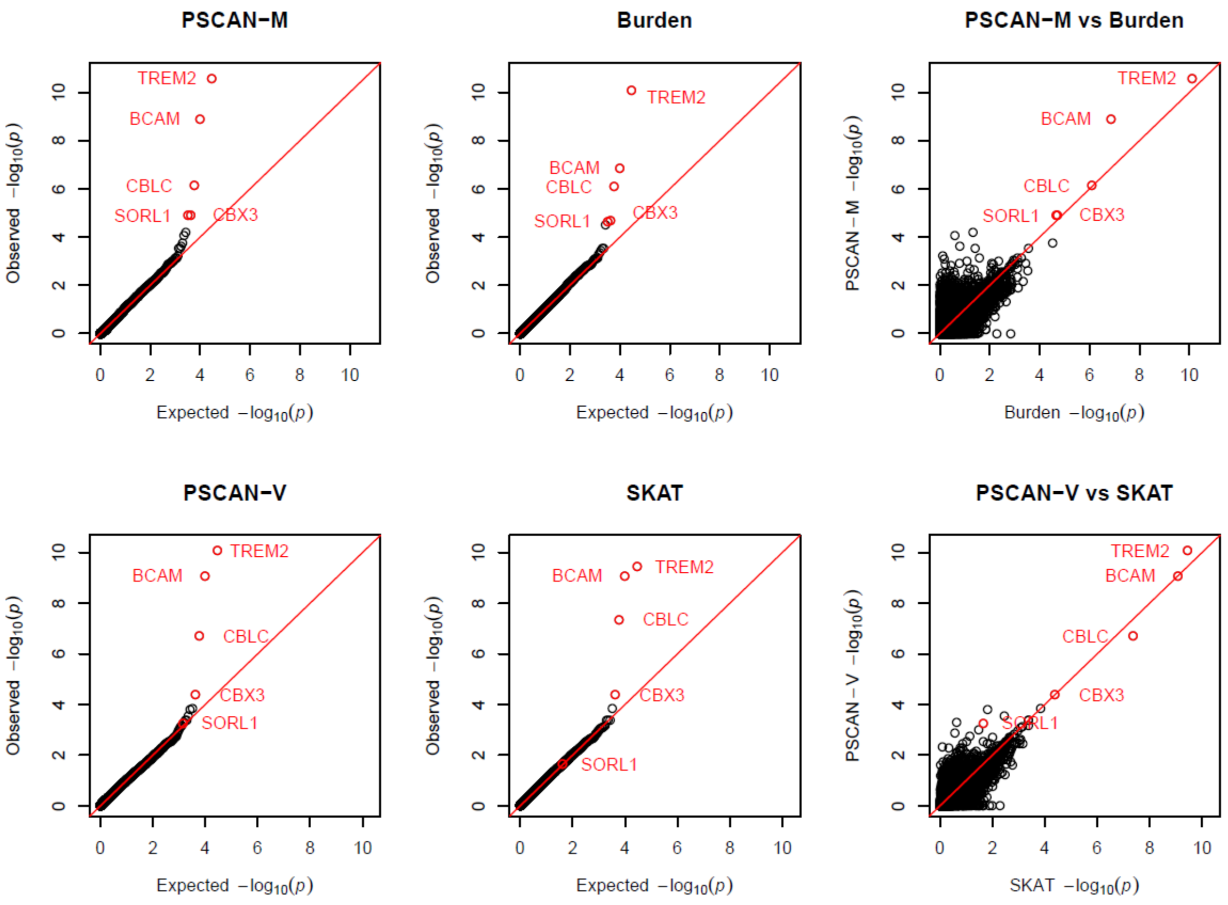

Fig. S7 Meta-analysis of the studies in ADSP for Alzheimer's disease. The left panel shows p-value QQ plots for the proposed tests (PSCAN-M or PSCAN-V); the middle panel shows the p-value QQ plots for the existing gene-level tests (Burden or SKAT); the right panel contains scatter plots comparing the PSCAN tests and the counterparts in existing methods. The genes significantly associated with AD in at least one test are highlighted in red.

Table S1: Identified signal regions in the ESP analysis of HDL and triglycerides. Chromosome positions of variants within each region are enclosed in parentheses with the p-value for the region in the front. For the PSCAN-M and SCAN1D-M, the p-value is from the burden test in the region; for the PSCAN-V and SCAN1D-V, the p-value is from the SKAT in the region.

Gene	Chr	PSCAN-M	PSCAN-V	SCAN1D-M	SCAN1D-V
CD36	7	$\mathbf{5 \times 10^{-8}}$ {80300449, 80301274}	$\mathbf{9 \times 10^{-8}}$ {80300449, 80301274}		$\mathbf{8 \times 10^{-8}}$ {80290383, 80290405, 80290459, 80290473, 80290474, 80290482, 80290494, 80290498, 80290500, 80290503, 80290527, 80290528, 80292318, 80292321, 80292325, 80292339, 80292400, 80292415, 80292417, 80292423, 80292426, 80292429, 80292448, 80293749, 80293761, 80293767, 80295758, 80295763, 80295802, 80295803, 80299280, 80299284, 80299332, 80300318, 80300346, 80300396, 80300400, 80300435, 80300439, 80300449, 80300472, 80300474, 80301274, 80301294, 80301310, 80301315, 80301330, 80301332}
CETP	16	$\mathbf{3 \times 10^{-8}}$ {56995905, 56995908, 56995935}	$\mathbf{1 \times 10^{-10}}$ {56995905, 56995908, 56995935}, $\mathbf{1 \times 10^{-3}}$ {57016092}	$\mathbf{5 \times 10^{-10}}$ {56995908, 56995935}	$\mathbf{1 \times 10^{-10}}$ {56995908, 56995935}, $\mathbf{1 \times 10^{-3}}$ {57016092}
NCK1	3		$\mathbf{6 \times 10^{-6}}$ {136646857, 136664533, 136664582, 136664604, 136664709, 136667116, 136667274, 136647045, 136664737, 136664745, 136664910, 136664970}		$\mathbf{4 \times 10^{-7}}$ {136664533, 136664582, 136664604, 136664709, 136664737, 136664745, 136664910}
PCSK7	11	$\mathbf{1 \times 10^{-7}}$ {117089205}	$\mathbf{1 \times 10^{-7}}$ {117089205}	$\mathbf{1 \times 10^{-7}}$ {117089205}	$\mathbf{1 \times 10^{-7}}$ {117089205}
APOC3	11	$\mathbf{4 \times 10^{-6}}$ {116701560, 116701613, 116703493, 116701284, 116701326, 116701353, 116701354}	$\mathbf{3 \times 10^{-6}}$ {116701560, 116701613, 116703493, 116701284, 116701326, 116701353, 116701354}	$\mathbf{6 \times 10^{-9}}$ {116701353, 116701354, 116701560, 116701613}	$\mathbf{3 \times 10^{-6}}$ {116701353, 116701354, 116701560, 116701613}
ARSI	5		$\mathbf{1 \times 10^{-6}}$ {149676860, 149676877, 149676959, 149677031, 149677103, 149677331, 149677472, 149677757, 149677982}		
CYP2C9	10	$\mathbf{6 \times 10^{-7}}$ {96701709, 96701758, 96701990, 96702011, 96702039, 96702047, 96702048, 96702066, 96702075, 96707569, 96707571, 96707587, 96707610, 96707691, 96708974, 96731871, 96740958, 96740981, 96740982, 96740988, 96741014, 96741053, 96741058, 96741066, 96741073, 96745820, 96745910, 96748636, 96748733, 96748777}		$\mathbf{5 \times 10^{-6}}$ {96698453, 96701709, 96701758, 96701990, 96702011, 96702039, 96702047, 96702048, 96702066, 96702075, 96707569, 96707571, 96707587, 96707610, 96707691, 96708974}	
NFATC1	18		$\mathbf{5 \times 10^{-8}}$ {77193611, 77193625, 77193713, 77208807, 77208845, 77211081}		$\mathbf{5 \times 10^{-8}}$ {77193581, 77193611, 77193625, 77193713, 77208807, 77208845, 77211081}
PGM1	1	$\mathbf{1 \times 10^{-5}}$ {64114301, 64120111, 64125260}	$\mathbf{5 \times 10^{-7}}$ {64114301, 64120111, 64125260}		

Table S2: Identified signal regions in the ADSP analyses. Chromosome positions of variants within each region are enclosed in parentheses with the p-value for the region in the front. For the PSCAN-M and SCAN1D-M, the p-value is from the burden test in the region; for the PSCAN-V and SCAN1D-V, the p-value is from the SKAT in the region.

Gene	Chr	PSCAN-M	PSCAN-V	SCAN1D-M	SCAN1D-V
BCAM	19	$\mathbf{6 \times 10^{-11}}${45316588}, $\mathbf{2 \times 10^{-8}}${45315445}	$\mathbf{6 \times 10^{-11}}${45316588}, $\mathbf{2 \times 10^{-8}}${45315445}	$\mathbf{4 \times 10^{-11}}${45316510, 45316525, 45316526, 45316553, 45316588}	$\mathbf{1 \times 10^{-10}}${45316510, 45316525, 45316526, 45316553, 45316588}
CBLC	19	$\mathbf{5 \times 10^{-8}}${45295664, 45295688, 45296767, 45296786, 45296806, 45297479, 45297525, 45303639}	$\mathbf{2 \times 10^{-8}}${45295664, 45295688, 45296767, 45296786, 45296806, 45297479, 45297525, 45303639}	$\mathbf{5 \times 10^{-9}}${45296767, 45296786, 45296806, 45297479, 45297525}	$\mathbf{8 \times 10^{-9}}${45296767, 45296786, 45296806, 45297479, 45297525}
CBX3	7	$\mathbf{8 \times 10^{-6}}${26242626, 26245994, 26248139, 26248148}	$\mathbf{4 \times 10^{-5}}${26242626, 26245994, 26248139, 26248148}	$\mathbf{4 \times 10^{-5}}${26245994, 26246035, 26248104, 26248139, 26248148}	$\mathbf{4 \times 10^{-5}}${26248104, 26248139}
SORL1	11	$\mathbf{1 \times 10^{-6}}${121340744, 121340776, 121340782, 121340794, 121348834, 121348842, 121358750, 121393359, 121393684, 121403213, 121403242, 121403248, 121403254, 121414293, 121414300, 121414334, 121414386, 121414390, 121414415, 121415993, 121416012, 121416045, 121416093, 121416096, 121416122, 121420682, 121420782, 121420791, 121421299, 121421313, 121421325, 121421341, 121421361, 121421364}	$\mathbf{8 \times 10^{-5}}${121393684}	$\mathbf{6 \times 10^{-7}}${121391383, 121391400, 121391401, 121391550, 121393359, 121393370, 121393684, 121403213, 121403242, 121403248, 121403254, 121414293, 121414300, 121414334, 121414386, 121414390, 121414415, 121415955, 121415993, 121416012, 121416045, 121416093, 121416096, 121416122, 121420682, 121420773, 121420782, 121420791, 121421299, 121421313, 121421325, 121421341, 121421361, 121421364, 121424660, 121424706, 121424741, 121424774, 121425897, 121425910, 121425939, 121425945, 121425968, 121425973, 121428095, 121428098, 121428101, 121429304, 121429334, 121429347, 121429424, 121429434, 121429469, 121429472, 121429476, 121429506, 121430256, 121430301, 121430312, 121430322}	$\mathbf{8 \times 10^{-5}}${121391383, 121391400, 121391401, 121391550, 121393359, 121393370, 121393684}
TREM2	6	$\mathbf{2 \times 10^{-12}}${41129252, 41129253}	$\mathbf{5 \times 10^{-12}}${41129252}	$\mathbf{1 \times 10^{-12}}${41127543, 41127561, 41127603, 41127605, 41127606, 41127615, 41127620, 41129078, 41129100, 41129105, 41129133, 41129188, 41129189, 41129195, 41129207, 41129208, 41129247, 41129252, 41129253, 41129264, 41129275, 41129277, 41129300, 41129309, 41129313, 41129345, 41129346}	$\mathbf{3 \times 10^{-10}}${41127543, 41127561, 41127603, 41127605, 41127606, 41127615, 41127620, 41129078, 41129100, 41129105, 41129133, 41129188, 41129189, 41129195, 41129207, 41129208, 41129247, 41129252, 41129253, 41129264, 41129275, 41129277, 41129300}
