# Supplementary material for: PSCAN: Spatial scan tests guided by protein structures improve complex disease gene discovery and signal variant detection
Source: Genome Biol. 2020 Aug 26;21:217. doi: 10.1186/s13059-020-02121-0 (PMC7448521; doi:10.1186/s13059-020-02121-0)
Supplement: Supplementary file 2 — Additional file 2 Review history. [file 13059_2020_2121_MOESM2_ESM.docx]

**Review history**

**First round of review**

**Reviewer 1**

In this paper, the authors proposed a scan test for two purposes: 1) evaluating gene level association signals and 2) pinpointing the specific region in the gene that drives the association signal for sequencing studies. The paper is well presented, with some interesting application.

1.     The algorithm for searching the causal region is confusing to me. Even though the windows defined through the protein structure can be overlapping, it seems that the algorithm will only choose a set of (nonoverlapping) windows as the final "region of association". It seems that if you have multiple windows that passed the significance threshold, and you will only choose the one with the smallest p-value if they overlap with each other. What is the rational behind this? Why not choose the union of these windows as all of them pass the significance threshold?

As in your example at the beginning of page 11,  suppose you have the same candidate sets: {4, 6}, {4, 6, 5}, {7, 8}, {7, 8, 11}, {2, 3, 4, 5, 6, 7, 8, 11}, which all passed the significance threshold. Imagine a situation that the most significant signal comes from {7, 8, 11},with the second significant signal comes from {2, 3, 4, 5, 6, 7, 8, 11}, with the third significant signal comes from {4, 6, 5}. From your algorithm, you will only be able to identify {7, 8, 11} and {4, 6, 5}, because {2, 3, 4, 5, 6, 7, 8, 11} is removed due to its overlap with {7, 8, 11}. This is counterintuitive.

Further, is there any theoretical guarantee about the final region that were chosen?

2.     I understand that the aim of this paper is to utilize the protein structure to improve hypothesis testing at DNA level. At GWAS and sequencing studies, many makers are within intron or other non-coding regions, promoter regions etc., which all have important biological significance, but have no protein 2D or 3D structure to map to. How would your model takes these SNPs into consideration? Also, the impact of synonymous SNPs?

The essence of this paper is to utilize external information to boost power for set-based association testing. In the ideal case, if your external information (for your case, the protein structure) indeed provides additional information, the method should be more powerful than methods that do not use additional information, such as SKAT. However, I believe that there are approaches that utilize other types of external information, such as the type of the SNPs. Would you also be able to compare your method with theirs (or at least comment on the relative advantages and disadvantages)?

3.     The Cauchy combination approach provides a faster and more elegant alternative compared to Monte Carlo approach for assessing statistical significance. I see that you evaluated the type I error at very small alpha levels, which is necessary for whole genome scan considering the total number of tests that need to be done. However, I am intrigued to know how Cauchy combination approach works with larger alpha levels (such as 0.05 or 0.01), as in a candidate gene analysis?  

4.     I have successfully downloaded and installed the package. A plus point on this. However, the manual is very simple, comparing to the complexity of the method. Because of the way that the protein structure is incorporated into the analysis, I can envision that the analysis is not as straightforward as methods that utilizes only the sequencing data. Especially, I think the challenges in using this package in fact lie in 1) how to get the data into the input format of the package, 2) how to obtain external information providing the protein structure information. A detailed vignette giving examples of running the function would be helpful.

**Reviewer 2**

In this manuscript, the authors propose PSCAN (protein-structure-based scan), an approach to evaluate gene-level associations with complex diseases and detect signal regions for protein-coding variants. In order to improve the power of gene-level association tests, the 3D protein structure is considered. The 3D coordinates of genetic variants in the protein space are obtained from the Protein Databank (PDB) and used to define the 3D windows in PSCAN. The spatial scan tests of both SKAT and burden are conducted on the overlapping 3D windows and the Cauchy method is used to combine multiple set-based p-values across windows. Besides, given a disease-associated gene, the signal regions within the gene are detected by a PSCAN search algorithm.

Simulation studies are conducted comparing PSCAN-M (for testing the mean effect) and PSCAN-V (for testing the variance effect) with burden, SKAT, and single variant (SV) tests. They show that generally PSCAN controls the type I error and can have higher power than the traditional SKAT and burden tests that do not take advantage of local clustering information. Besides, the PSCAN methods also can better identify (have higher sensitivity) signal regions relative to the SV analysis, when the variants are rare. Two exome sequencing datasets are used in the applications, the NHLBI ESP data for lipid traits and the ADSP data for Alzheimer's disease.

This is in principle a useful, although somewhat straightforward, extension of the existing gene-based tests to incorporate 3D protein structure. I would have liked to see a more detailed analysis/discussion of the effect of uncertain 3D structures on the power of the tests. The authors should also consider comparing with 1D scanning in order to assess how useful the 3D structure is beyond the 1D scanning.

The paper is easy to follow but there are several details missing that can confuse the reader.  Specifically, I have the following points for the authors to consider:

On page 7, when defining the windows for variants in the 3D protein space, the authors define different graphs depending on a window size w. However, it is unclear how they determine the window sizes w for the 3D Euclidean distance.

The authors provide an example with m=12 variants. The example generates 12  graphs with 2×m-1=23 different windows. Do the authors recommend generating 2×m-1 windows and stop with the graph that contains all the variants?

    On pages 6-7, the authors also mention that other 3D windows can be considered in traditional scan tests with pre-specified shapes (spherical, rectangular, etc). Please provide more details on the traditional scan tests, for example, adding some references and demonstrate why the number of 3D windows in the traditional scan tests is large compared with the proposed approach.

On pages 9-10, the authors mention they use the Cauchy combination method to combine p values across all windows, but in the Methods section on page 21 they seem to mention a Monte Carlo simulation approach. Please clarify.

In simulations and the exome-sequencing applications, the authors compare the PSCAN tests with traditional SKAT and burden tests. However, it seems unfair to compare the spatial scan tests with non-scan tests. Why don't the authors compare the PSCAN with 1D scan tests? One can easily implement such 1D scan tests. Using linear scan tests as competitors of the 3D scan tests seems more reasonable than just conducting the traditional SKAT and burden tests that suffer from power issues when many background variants are included.

    For the ESP analyses, do the authors focus on missense, rare variants in the SKAT/burden tests? Can the authors add more details on the specific analyses? These details are important in evaluating the performance…

Looking at the results in Tables 2&3, the advantage of the PSCAN seems to mostly come from testing small windows with only a few variants. How far away are those variants in the 1D space and how much does it help to have the 3D protein structure?

In page 12, the simulation setting, the authors simulate the 3D coordinates of causal variants in the protein space using normal distribution. What is the mean of the normal distribution? Please clarify.

**Response to reviewers**

**Reviewer #1:**

*In this paper, the authors proposed a scan test for two purposes: 1) evaluating gene level*

*association signals and 2) pinpointing the specific region in the gene that drives the association*

*signal for sequencing studies. The paper is well presented, with some interesting application.*

Thank you!

*1. The algorithm for searching the causal region is confusing to me. Even though the windows*

*defined through the protein structure can be overlapping, it seems that the algorithm will only*

*choose a set of (nonoverlapping) windows as the final "region of association". It seems that if*

*you have multiple windows that passed the significance threshold, and you will only choose the*

*one with the smallest p-value if they overlap with each other. What is the rational behind this?*

*Why not choose the union of these windows as all of them pass the significance threshold?*

*As in your example at the beginning of page 11, suppose you have the same candidate sets: {4,*

*6}, {4, 6, 5}, {7, 8}, {7, 8, 11}, {2, 3, 4, 5, 6, 7, 8, 11}, which all passed the significance*

*threshold. Imagine a situation that the most significant signal comes from {7, 8, 11}, with the*

*second significant signal comes from {2, 3, 4, 5, 6, 7, 8, 11}, with the third significant signal*

*comes from {4, 6, 5}. From your algorithm, you will only be able to identify {7, 8, 11} and {4, 6,*

*5}, because {2, 3, 4, 5, 6, 7, 8, 11} is removed due to its overlap with {7, 8, 11}. This is*

*counterintuitive.*

*Further, is there any theoretical guarantee about the final region that were chosen?*

**Response:** We chose the nonoverlapping-window approach over the union-window

approach described by the Reviewer based on both theoretical and practical considerations.

Signal region identification procedures in 1D scan methods have been studied theoretically

in previous research (e.g. [1-3]). When signals are sufficiently strong and signal regions

are well separated, the nonoverlapping-window procedure can achieve asymptotic

optimality (i.e. in terms of reliably separating the true signal region from noise) [1,3]. For

example, suppose there is a cluster of signal SNPs in a gene and these SNPs cumulatively

have a large effect so that many larger windows overlapping with the signal region pass

the significance threshold. Then, the union-window approach would pick many neutral

SNPs as signals in addition to the signal SNPs. The nonoverlapping-window approach

would not include these neutral SNPs, because the smaller window around the causal SNPs

would tend to have a more significant p-value and any overlapping windows would be

removed.

An alternative signal region identification procedure has been developed to deal with the

situation where signals are relatively weak and/or signal regions are possibly nested [2,4].

This procedure only removes windows that overlap by more than the pre-specified overlap

fraction *f* (see Algorithm S1 in the Supplementary File). When *f=1*, this algorithm is

essentially the union-window approach. Although our paper focuses on the

nonoverlapping-window approach, we think the alternative approach could still be useful

to some users in practice. Therefore, we implemented it in our software package and allow

users to specify a parameter *f* for the overlap fraction (by default, *f=0*, perform

nonoverlapping-window search).

The clarification has been added into the revised paper. Please see the Results subsection

“PSCAN search algorithm for signal regions” (on page 7) and the Supplementary File

(page 1).

[1] Jeng, X. J., Cai, T. T., and Li, H. (2010). Optimal sparse segment identification with application in copy number

variation analysis. Journal of the American Statistical Association, 105(491):1156-1166.

[2] Zhang, N. R., Siegmund, D. O., Ji, H., and Li, J. Z. (2010). Detecting simultaneous changepoints in multiple

sequences. Biometrika, 97(3):631-645.

[3] Li, Z., Liu, Y. & Lin, X. (2019). Simultaneous detection of signal regions with applications in genome-wide

association studies. Preprint at https://arxiv.org/abs/1710.05021.

[4] Li, Z., et al. (2019) "Dynamic scan procedure for detecting rare-variant association regions in whole-genome

sequencing studies." The American Journal of Human Genetics 104.5: 802-814.

*2. I understand that the aim of this paper is to utilize the protein structure to improve*

*hypothesis testing at DNA level. At GWAS and sequencing studies, many makers are within*

*intron or other non-coding regions, promoter regions etc., which all have important biological*

*significance, but have no protein 2D or 3D structure to map to. How would your model takes*

*these SNPs into consideration? Also, the impact of synonymous SNPs?*

**Response:** Our paper focuses on protein-coding variants. The power of existing association

analysis methods is still limited by the high background rate of neutral variants even when

only analyzing protein-coding variants. It would be possible to analyze non-coding variants

in our framework by creating an additional window for them, as for coding variants without

structural information. Another way that our method could potentially be useful for

identifying causal non-coding variants is by leveraging 3D chromatin structures inferred

from HiChIP, ChIA-PET, and related technologies. However, such integration of coding

and non-coding variants would require extensive development and evaluation and is

beyond the scope of this study.

Finally, synonymous variants can be included in our structure-guided tests via their 3D

locations in protein structure. However, while synonymous variants can have functions,

e.g. via influencing mRNA transport or translational efficiency, these are not mediated

through changes in amino acid sequence or protein 3D structure. Indeed, our previous work

has shown that synonymous variants do not exhibit any detectable spatial constraint [5].

Thus, since the vast majority of synonymous variants are unlikely to be causal, we do not

consider them here to avoid reducing power.

We have added comments about synonymous variants in the Results subsection “Mapping

variants into 3D protein space” (on page 4) and about these possible extensions in the last

paragraph of the Discussion section (on pages 13-14).

[5] Sivley, R. M., Dou, X., Meiler, J., Bush, W. S., & Capra, J. A. (2018). Comprehensive analysis of constraint on the

spatial distribution of missense variants in human protein structures. The American Journal of Human Genetics, 102(3),

415-426.

*The essence of this paper is to utilize external information to boost power for set-based*

*association testing. In the ideal case, if your external information (for your case, the protein*

*structure) indeed provides additional information, the method should be more powerful than*

*methods that do not use additional information, such as SKAT. However, I believe that there are*

*approaches that utilize other types of external information, such as the type of the SNPs. Would*

*you also be able to compare your method with theirs (or at least comment on the relative*

*advantages and disadvantages)?*

**Response:** Other variant annotations could be readily integrated with structural

information in our PSCAN framework. For example, variants could be weighted based on

their evolutionary conservation or predicted effect on protein structure and stability.

Integrating functional annotations as variant weights has improved power in traditional

SNP-set tests. For genes with no protein structural data, we suggest integrating other

potentially informative variant annotations. We have added discussion of the incorporation

of additional annotations in the Discussion section (on pages 13-14). However, since there

is not an established standard against which to benchmark our method, we have not

explored this dimension further.

*3. The Cauchy combination approach provides a faster and more elegant alternative*

*compared to Monte Carlo approach for assessing statistical significance. I see that you*

*evaluated the type I error at very small alpha levels, which is necessary for whole genome scan*

*considering the total number of tests that need to be done. However, I am intrigued to know how*

*Cauchy combination approach works with larger alpha levels (such as 0.05 or 0.01), as in a*

*candidate gene analysis?*

**Response:** Thank you for bringing up this important point. We can imagine that some

PSCAN users may be interested in testing only a handful of candidate genes. The empirical

type I error rate is 0.055 for PSCAN-Mand 0.052 for PSCAN-V at alpha level of 0.05; and

0.011 and 0.010 at alpha level of 0.01. As reported by previous papers [6,7], the Cauchy

p-value combination method properly controls the type I error rate for small p-value

(<10−4) but can have a slight inflation in the type I error for larger p-values. Therefore, in

the candidate gene analyses, using Monte Carlo simulation to evaluate the significance of

the minimal p-value statistic is more robust than the Cauchy method. We have added this

comment in the Methods subsection “Minimum p-value scan statistic” (on page 15).

[6] Liu Y, Xie J. Cauchy Combination Test: A powerful test with analytic p-value calculation under arbitrary

dependency structures. J Am Stat Assoc. 2019;0(0):1-18.

[7] Liu Y, Chen S, Li Z, Morrison AC, Boerwinkle E, Lin X. ACAT: A fast and powerful p value combination method

for rare-variant analysis in sequencing studies. Am J Hum Genet. 2019;104(3):410-421.

*4. I have successfully downloaded and installed the package. A plus point on this. However,*

*the manual is very simple, comparing to the complexity of the method. Because of the way that*

*the protein structure is incorporated into the analysis, I can envision that the analysis is not as*

*straightforward as methods that utilizes only the sequencing data. Especially, I think the*

*challenges in using this package in fact lie in 1) how to get the data into the input format of the*

*package, 2) how to obtain external information providing the protein structure information. A*

*detailed vignette giving examples of running the function would be helpful.*

**Response:** We have provided a detailed vignette giving examples of running the function

and explaining how the software addresses the two challenges. Please see the updated

PSCAN package at <https://tangzheng1.github.io/tanglab/software.html>

**Reviewer #2:**

*This is in principle a useful, although somewhat straightforward, extension of the existing genebased*

*tests to incorporate 3D protein structure. I would have liked to see a more detailed*

*analysis/discussion of the effect of uncertain 3D structures on the power of the tests.*

**Response:** Thank you for your comment. Given that computationally derived structural

models, which are often lower resolution than experimentally derived structures, are

available for more than 80% of human proteins, this is a particularly important point. As

described in more detail in the response below, our window definition strategy allows for

uncertainty in protein structure. As long as the connected components in the graphs of

variants defined based on their pairwise distances are maintained, then the resulting

windows would be the same between two structures of different resolution. Given that the

distances observed between pairs of variants are much greater on average (~26 Å) than the

resolution of even the lowest resolution models (~8 Å), the uncertainty should rarely

influence the windows evaluated.

To evaluate this, we compared windows and test results for proteins with both experimental

and computational structural models in the ESP analysis. In this analysis, on average 95%

of the windows defined using experimentally determined structures were recovered using

the less accurate computationally predicted structures. Furthermore, the results of

association studies are extremely similar between experimental and computational

structures (p-value Pearson correlation of 0.99; Figure S6). Therefore, our method is robust

to realistic levels of uncertainty in the protein structural models. We have added this

clarification in the subsection “Computationally predicted structures are sufficiently

accurate for PSCAN” (on page 11).

*The authors should also consider comparing with 1D scanning in order to assess how useful the*

*3D structure is beyond the 1D scanning.*

**Response:** Thank you for the suggestion. We have compared PSCAN with 1D scan

methods. **Please see the detailed response below.**

*The paper is easy to follow but there are several details missing that can confuse the*

*reader. Specifically, I have the following points for the authors to consider:*

*On page 7, when defining the windows for variants in the 3D protein space, the authors define*

*different graphs depending on a window size w. However, it is unclear how they determine the*

*window sizes w for the 3D Euclidean distance.*

*The authors provide an example with m=12 variants. The example generates 12 graphs with*

*2×m-1=23 different windows. Do the authors recommend generating 2×m-1 windows and stop*

*with the graph that contains all the variants?*

**Response:** Thank you for sending this important question. Our windowing procedure does

not require any decision on the part of the user. It considers all 2 × 𝑚𝑚 − 1 possible

windows. For a given window size 𝑤𝑤, we connect all pairs of nodes with distance less than

𝑤𝑤 and define windows based on the resulting graph. Even though the size 𝑤𝑤 can take an

infinite number of values, the number of possible graphs is limited because the graph 𝐺𝐺(𝑤𝑤)remains the same when 𝑤𝑤 is between two consecutive numbers in the sorted list of pairwise distances. In the example, we have 12 nodes and 66 pairwise distances (denoted by 𝑑𝑑𝑖𝑖𝑖𝑖 in the paper, where 𝑖𝑖, 𝑗𝑗 are the 𝑖𝑖𝑡𝑡ℎ, 𝑗𝑗𝑡𝑡ℎ nodes). The distance between 7𝑡𝑡ℎ and 8𝑡𝑡ℎ node (i.e. 𝑑𝑑78) is the smallest, so any value of 𝑤𝑤 < 𝑑𝑑78 will end up with the same graph. This is represented in the first graph in Figure 1 where no nodes are connected and we have 12 windows each of which contains one node. In other words, none of nodes are connected in the graph, so they belong to separate windows. Once the value of 𝑤𝑤 becomes ≥ 𝑑𝑑78 and < 𝑑𝑑46 (the second smallest pairwise distance), we will have the second graph in which the window {7,8} is added to the window set. This procedure finishes when the value of 𝑤𝑤 is greater than the greatest pairwise distance between nodes. This results in the graph in which all the nodes can reach each other, and the final window including all the nodes is added to the window set (the last graph in Figure 1). Thus, the total number of windows (2 × 𝑚𝑚 − 1) is not determined subjectively, but by this scan procedure. We have rewritten several paragraphs in the subsection “Defining flexibly shaped windows in 3D protein space” to clarify (on page 5).

*On pages 6-7, the authors also mention that other 3D windows can be considered in traditional*

*scan tests with pre-specified shapes (spherical, rectangular, etc). Please provide more details on*

*the traditional scan tests, for example, adding some references and demonstrate why the number*

*of 3D windows in the traditional scan tests is large compared with the proposed approach.*

**Response:** We have added Figure S1 to demonstrate the definition of windows in the

traditional scan approach using spherical regions. In this example with 12 nodes, our

PSCAN approach generates 23 windows, but the traditional approach generates 87

windows.

We have also provided more details on how the traditional spatial scan tests are different

from our scan test and included references [1-4] for traditional scan tests. Please see the

clarification in the subsection “Defining flexibly shaped windows in 3D protein space” (on

page 5).

[1] Kulldorff M, Nagarwalla N. Spatial disease clusters: detection and inference. Stat Med. 1995;14(8):799-810.

[2] Kulldorff M. A spatial scan statistic. Commun Stat Theory Methods.1997;26(6):1481-1496.

[3] Kulldorff M, Huang L, Pickle L, Duczmal L. An elliptic spatial scan statistic. Stat Med. 2006;25(22):3929{3943.

[4] Naus J. Clustering of random points in two dimensions. Biometrika. 1965;52(1-2):263-266.

*On pages 9-10, the authors mention they use the Cauchy combination method to combine p*

*values across all windows, but in the Methods section on page 21 they seem to mention a Monte*

*Carlo simulation approach. Please clarify.*

**Response:** Both the Cauchy method and Monte Carlo simulation can be used to test gene level association. Our paper focuses on using the Cauchy method because it is

computationally fast and previous research has shown that it can properly control the type

I error regardless of correlations of individual tests when p-values are small (<10−4, as is

the significance level in the genome-wide analysis) [5,6]. However, the Cauchy method

can have a slight inflation in the type I error for large p-values. Therefore, in the candidate

gene analysis where only a handful of genes are tested, we use the more robustMonte Carlo

simulation approach to evaluate the significance of scan statistics. We have modified the

Methods section (on page 15) to clarify this point.

[5] Liu Y, Xie J. Cauchy Combination Test: A powerful test with analytic p-value calculation under arbitrary dependency

structures. J Am Stat Assoc. 2019;0(0):1-18.

[6] Liu Y, Chen S, Li Z, Morrison AC, Boerwinkle E, Lin X. ACAT: A fast and powerful p value combination method

for rare-variant analysis in sequencing studies. Am J Hum Genet. 2019;104(3):410-421.

*In simulations and the exome-sequencing applications, the authors compare the PSCAN tests*

*with traditional SKAT and burden tests. However, it seems unfair to compare the spatial scan*

*tests with non-scan tests. Why don't the authors compare the PSCAN with 1D scan tests? One*

*can easily implement such 1D scan tests. Using linear scan tests as competitors of the 3D scan*

*tests seems more reasonable than just conducting the traditional SKAT and burden tests that*

*suffer from power issues when many background variants are included.*

**Response:** Thank you for your suggestion. We compared PSCAN with burden and SKAT

because these are the most commonly used gene-level association tests. In the revision, we

have implemented the 1D scan gene-level test and signal detection (see the description of

this approach “SCAN1D method” on page 16). We have compared PSCAN with SCAN1D

in the simulation studies and real data analyses (results in Figures 3 and 4, Tables 2 and 3,

and the relevant text in the revised paper, pages 8-12). In the simulation studies, the PSCAN

methods outperform SCAN1D methods in almost all scenarios. The SCAN1D methods are

more powerful than burden and SKAT test when signal is sparse. However, the situation

reverses when the signal is dense and unidirectional. In this case, the most effective

window is often the whole gene region and SCAN1D tests are less effective because they

have to pay high penalty for testing many windows. (PSCAN does not suffer from this

limitation as it considers a much smaller number of windows guided by the shape of the

protein itself.) In analyses of real data, we also demonstrate the advantage of PSCAN over

SCAN1D. In particular, overall, PSCAN gene-level tests produced more significant pvalues

compared to their SCAN1D counterparts (Tables 2 and 3). Furthermore, in the

several examples, we highlight how PSCAN identifies signal windows of variants that are

distant along the chromosome (e.g., Figure 6, Tables S1 and S2).

*For the ESP analyses, do the authors focus on missense, rare variants in the SKAT/burden tests?*

*Can the authors add more details on the specific analyses? These details are important in*

*evaluating the performance…*

**Response:** On page 9, we have clarified that all non-synonymous protein-coding variants

(common and rare) were included in ESP data analyses. All methods analyzed the same set

of variants. Many details on the ESP data analyses have been presented in the Methods

subsection “NHLBI ESP data and analyses” on page 17. For the ADSP analyses, we

focused on missense variants with MAF < 0.05 to compare with previous ADSP work and

highlighted the utility for rare variant analyses.

*Looking at the results in Tables 2&3, the advantage of the PSCAN seems to mostly come from*

*testing small windows with only a few variants. How far away are those variants in the 1D space*

*and how much does it help to have the 3D protein structure?*

**Response:** Thank you for the question. We now present the chromosome position

information for the signal variants in Table S1 and S2, and we demonstrate in detail how

protein structure helps in the case of three example genes (CD36, PGM1 in ESP and SORL1

in ADSP) in the revised paper (on pages 10, 11, and 12).

*In page 12, the simulation setting, the authors simulate the 3D coordinates of causal variants in*

*the protein space using normal distribution. What is the mean of the normal distribution? Please*

*clarify.*

**Response:** We have clarified on page 8 that the coordinates for the neutral variants were

sampled from a standard normal distribution and the coordinates for the causal variants

were sampled from a zero-mean normal distribution with the standard deviation ρ. Note

that the mean values do not affect the relative positions of those variants as long as neutral

and causal variants have the same mean value. We considered different values of ρ to

mimic different dispersion levels of causal variants relative to the neutral ones.

**Second round of review**

**Reviewer 1**

The authors have addressed my previous questions.

**Reviewer 2**

The authors have addressed all my comments.
